# Metabolic Dynamics in Short- and Long-Term Microgravity in Human Primary Macrophages

**DOI:** 10.3390/ijms22136752

**Published:** 2021-06-23

**Authors:** Cora S. Thiel, Christian Vahlensieck, Timothy Bradley, Svantje Tauber, Martin Lehmann, Oliver Ullrich

**Affiliations:** 1 Institute of Anatomy, Faculty of Medicine, University of Zurich, Winterthurerstrasse 190, 8057 Zurich, Switzerland; christian.vahlensieck@uzh.ch (C.V.); timothy.bradley@uzh.ch (T.B.); svantje.tauber@uzh.ch (S.T.); 2 Innovation Cluster Space and Aviation (UZH Space Hub), Air Force Center, University of Zurich, Überlandstrasse 271, 8600 Dübendorf, Switzerland; 3 Biocenter LMU Muenchen, Department of Biology I–Botany, Großhaderner Strasse 2–4, 82152 Planegg-Martinsried, Germany; martin.lehmann@biologie.uni-muenchen.de; 4 Space Biotechnology, Department of Machine Design, Engineering Design and Product Development, Institute of Mechanical Engineering, Otto-von-Guericke-University Magdeburg, Universitätsplatz 2, 39106 Magdeburg, Germany; 5 Space Medicine, Ernst-Abbe-Hochschule (EAH) Jena, Department of Industrial Engineering, Carl-Zeiss-Promenade 2, 07745 Jena, Germany; 6 Zurich Center for Integrative Human Physiology (ZIHP), University of Zurich, Winterthurerstrasse 190, 8057 Zurich, Switzerland; 7 Space Life Sciences Laboratory (SLSL), Kennedy Space Center (KSC), 505 Odyssey Way, Exploration Park, FL 32953, USA

**Keywords:** spaceflight, sounding rocket, microgravity, metabolomics, immune cells

## Abstract

Microgravity acts on cellular systems on several levels. Cells of the immune system especially react rapidly to changes in gravity. In this study, we performed a correlative metabolomics analysis on short-term and long-term microgravity effects on primary human macrophages. We could detect an increased amino acid concentration after five minutes of altered gravity, that was inverted after 11 days of microgravity. The amino acids that reacted the most to changes in gravity were tightly clustered. The observed effects indicated protein degradation processes in microgravity. Further, glucogenic and ketogenic amino acids were further degraded to Glucose and Ketoleucine. The latter is robustly accumulated in short-term and long-term microgravity but not in hypergravity. We detected highly dynamic and also robust adaptative metabolic changes in altered gravity. Metabolomic studies could contribute significantly to the understanding of gravity-induced integrative effects in human cells.

## 1. Introduction

Since the early days of spaceflight, it has been known that human cell systems react to microgravity stimulation both in situ but also isolated from the human body in vitro [1,2,3,4,5]. Experiments with C57BL/6J mice on the Space Shuttle Atlantis mission STS-135 demonstrated an altered metabolic homeostasis [6] and raised the question of spaceflight-induced immune dysfunction being linked to systemic metabolic changes [7]. Ultimately, studying the metabolic effects of microgravity is relevant for astronaut safety and performance [8]. Recent studies have demonstrated that reactions of human immune cells to altered gravity do not require prolonged exposure, for example on orbital space stations, but that initial reactions occur after seconds of microgravity or hypergravity, as demonstrated by the oxidative burst reaction in macrophages during parabolic flight [9] and International Space Station (ISS) experiments [10].

Since then, several studies have been performed which have uncovered a plethora of effects, including responses to altered gravity on the transcriptional level. Transcriptomic responses have been observed already after 20 s of altered gravity [11,12,13], as well as impacts on cell cycle regulation [14], differential expression of microRNA [15], changed levels of chromatin regulators after minutes of microgravity [16], and long-term stability of cells on the ISS over the course of 11 days without major morphologic alterations despite de-fucosylated membrane proteins [17] (reviewed in [18]). However, the underlying molecular mechanisms of gravitational sensing and consecutively triggered reactions remain unknown.

Cellular mechanisms typically involve multiple types of molecules and therefore operate on several levels, including the genome and its conformation, the transcriptome, the proteome, the metabolome, and the ionome [19]. Metabolomics, as an emergent omics discipline that has gained traction in the last 10 years [20], is regarded to be especially sensitive. Small external or internal stimuli can lead to rapidly acting strong effects that, in contrast to transcriptomics or genomics, exceed input signals by several orders of magnitude (up to 100–10,000× for disease-related metabolic markers, in contrast to 1–10× for protein markers) [21]. The sensitivity of the metabolome to small external stimuli suggests that gravitational changes could also represent such stimuli. This sensitivity of the metabolome to external stimuli has recently contributed to the successful application of metabolomics in multiple biological research fields: in plant physiology, *Arabidopsis thaliana* was proven to bear a rapid heat shock response system, that includes salicylic acid as an early stress response factor with peak intensity increased several-fold only five minutes after onset of heat stimulation, requiring rapid reaction times. Other heat shock response factors emerged after 4 h, and some cold shock response factors only after 4 days, exemplifying the large potential spread of metabolic reaction times [22,23]. For *Drosophila melanogaster*, a nutrient stress assay could recently demonstrate a fundamental impact on systemic metabolic processes 1 h after feeding starved flies [24]. Vasilakou et al. measured an adaptation of decreased glycolytic metabolite intensity as a reaction to nutrient stress within only 10 s in *Escherichia. coli* [25]. These studies demonstrate that changes in the metabolome occur over a wide range of time spans, rendering metabolomics a potentially interesting technique to study the reaction of cells to altered gravity.

So far, only a handful of studies have been conducted on the metabolic effects of microgravity and hypergravity. The prominent NASA twin study included an untargeted metabolomic screen of blood plasma and a targeted urine screen. In the plasma compartment, genotoxicity- and inflammation-related metabolites and amino acid levels were significantly altered [26]. In another recent study, isolated cells were exposed to 11 days of microgravity onboard the ISS during the CELLBOX-PRIME mission. In this study, 8 metabolites showed significantly altered supernatant concentrations, including increased levels of Fucose, which could be explained by cell surface de-fucosylation [17]. Further, a few metabolomics studies in the field have been performed on ground-based facilities. For example, human bone-derived primary osteoblasts—representing one of the most-affected tissues in microgravity—react to vector-averaged gravity by a stimulated glycolysis and pentose phosphate metabolism but an interrupted Krebs cycle [27]. Further, a human hepatic cell line and human biliary tree stem/progenitor cells have been shown to increase their glycolytic metabolism under vector-averaged gravity [28].

These studies showed a significant influence of microgravity or vector-averaged gravity on the metabolome. A proposed next step in the metabolic research field are correlation studies between different platforms, focusing on the known property of metabolites to respond to perturbations in clusters and not as independent metabolites [29].

To gain insights into the underlying gravity-dependent mechanisms, we applied metabolomics analyses to two experiments performed on different platforms: a suborbital ballistic rocket flight and an ISS microgravity experiment. This comparative study is focused on shared metabolites and shared metabolite clusters between the two platforms, so that effects that are conserved between single experiments can be identified.

## 2. Results

**Figure 1:**
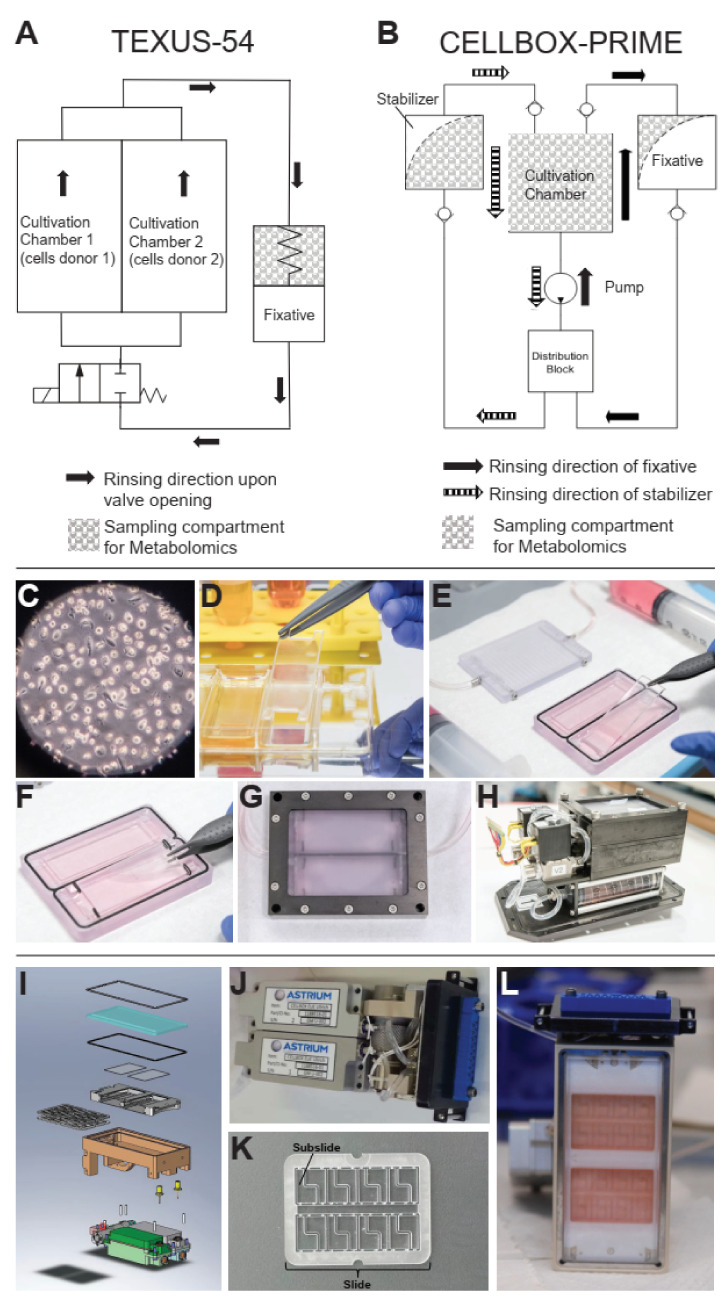
The experimental setups and fixation mechanisms of the TEXUS-54 FLUFIX hardware and the CELLBOX-PRIME hardware. (**A**) The layout of the FLUFIX hardware. Primary macrophages are stored inside two parallel cultivation chambers. Fixation solution is stored in a spring-loaded tank that injects its content into the two cultivation chambers upon valve release. Hereby, the cultivation chamber supernatant is flushed into the fixative container back chamber and thereby replaced by fixative. Metabolomics samples are gathered from the fixative container back chamber. Adapted from [17]. (**B**) The layout of the CELLBOX-PRIME hardware. The cell cultivation chamber is connected to two tanks, a fixative and a stabilizer container. First, the fixative is flushed into the cultivation chamber by the pump, thereby (partly) displacing the cell supernatant into the fixative tank back chamber. After fixation, the stabilizer is flushed into the cultivation chamber by the pump, partly displacing the cultivation chamber supernatant into the stabilizer container back chamber. Chambers that are highlighted in grey are used as metabolomics samples. (**C**) Human M1 Macrophages were used for both studies, displayed through a microscope. (**D**–**H**) TEXUS-54 hardware (**D**) Preparation of the slide containing the adherent macrophages. (**E**) Insertion of the bottom slide into the cultivation chamber. (**F**) Insertion of the top slide into the cultivation chamber. (**G**) Casing of the cultivation chamber. (**H**) Final assembly of the hardware unit. (**I**–**L**) CELLBOX-PRIME hardware, adapted from [17]. (**I**) Technical blueprint of the flight hardware with the two tanks and the pump at the bottom, casing, experimental chamber, and cover on top. (**J**) Tanks and hardware. (**K**) Experimental slide, compartmented into subslides. L Filled slides, carrying macrophages in media.

This study aimed at comparatively assessing the effects of short-term and long-term microgravity on the supernatant metabolome of primary human macrophage cultures. Therefore, two datasets were acquired on different microgravity platforms. In the TEXUS-54 FLUFIX experiment, macrophages were grown on macrolon slides and launched with a suborbital rocket during the TEXUS-54 flight campaign, fixed automatically during the flight using a system of chambers (Figure 1A,C–H), and the supernatants were collected. During the CELLBOX-PRIME mission, macrophages on compartmented macrolon slides were placed in automated flight hardware and launched to the ISS where they were automatically fixed and stabilized and the supernatants collected (Figure 1B,I–L).

**Figure 2:**
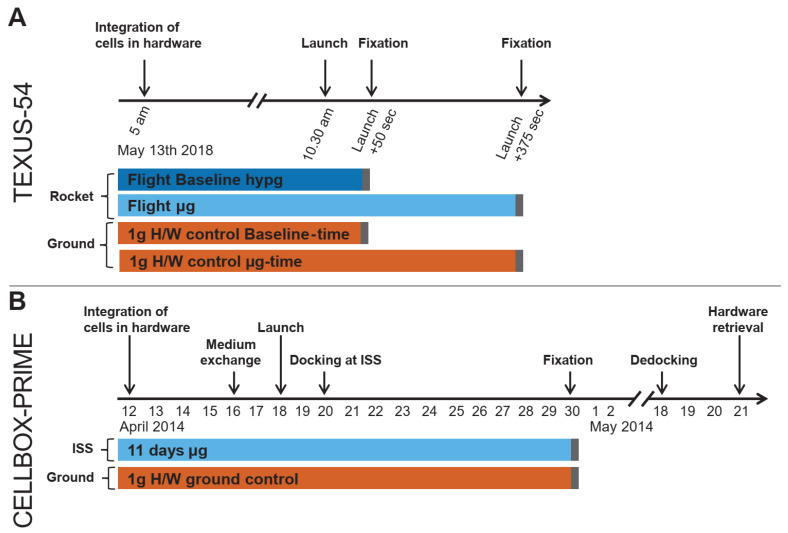
Experimental fixation scheme of the two campaigns. (**A**) The macrophages were separated and integrated into the TEXUS-54 hardware 5.5 h prior to launch. The rocket was launched at 10:30. After 50 s, the first hypergravity (hypg) sample was fixed. Then, 375 s after launch, the microgravity (µg) sample was fixed. Two ground controls that were integrated into the hardware but not launched were taken at the corresponding timepoints of the different fixation times. (**B**) The CELLBOX-PRIME hardware was uplifted to the ISS. After 11 days in microgravity, cells were fixed and stabilized, thereby stopping biological activity. After another 19 days, samples were recovered and prepared for analysis. Corresponding ground controls that were integrated into the same hardware and that were exposed to the same temperature profile as the flight samples were fixed at the same dates.

The TEXUS-54 campaign comprised four samples, hypergravity (hypg), microgravity (µg) and two ground controls, taken at corresponding timepoints according to the flight samples (Figure 2A). After launch, both flight samples were exposed to 50 s of hypergravity (acceleration phase of the rocket), and the microgravity sample to an additional 325 s of microgravity (consecutive ballistic flight) before fixation. Consequently, the µg sample was initially exposed to hypergravity prior to the µg phase. Therefore, technically it is an altered gravity sample with a dominant fraction of microgravity exposure time (87%). To be able to distinguish between hypergravity and microgravity effects, the hypergravity sample served also as a baseline representing a reference point to identify effects that were already caused by the hypergravity exposure. The CELLBOX-PRIME experiment consisted of five microgravity flight samples, with 11 days of microgravity onboard of the ISS and three corresponding ground control samples (Figure 2B).

**Figure 3:**
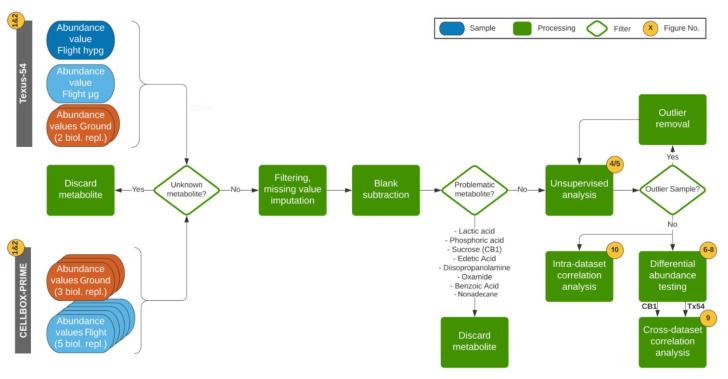
Workflow schematics of the data analysis. Corresponding figures from this paper are indicated in yellow circles. Samples that were analyzed by Gas Chromatography - Mass Spectrometry (GC-MS) and were peak-called, standardized and-post processed per experiment, were analyzed as abundance values. Metabolite spectra that could not be matched with a well-characterized reference metabolite were therefore called unknown metabolites and were filtered out from the datasets. Consecutively, metabolites with a large number of missing values/non-present metabolites per dataset were excluded, and metabolites with a small number of missing values underwent missing value imputation. Biological media blanks were subtracted. Further, problematic metabolites that were impurities from the equipment, used for fixation or with no biological information yield, were discarded. An unsupervised analysis (Figure 4 and Figure 5), based on heatmaps and principal component analysis, was performed and potential outlier samples removed. Consecutively, metabolites that were significantly differentially abundant in different experimental groups were identified per experiment (Figure 6, Figure 7 and Figure 8). Metabolites that were present in both datasets were compared in terms of fold change and direction of regulation (Figure 9). Independently, intra-dataset metabolite abundance values were correlated against each other to be able to detect potential regulatory associations (Figure 10).

The generated samples were supernatants of the cell cultures. After metabolite analysis using a gas chromatography–mass spectrometry (GC-MS) platform, the specific medium background (blank) was subtracted from the data to obtain metabolite uptake and secretion profiles. Consecutively, the two datasets were separately preprocessed and assessed for parallel or orthogonal differences. The detailed workflow is shown in Figure 3.

### 2.1. Identification of Metabolic Effects in Altered Gravity

**Figure 4:**
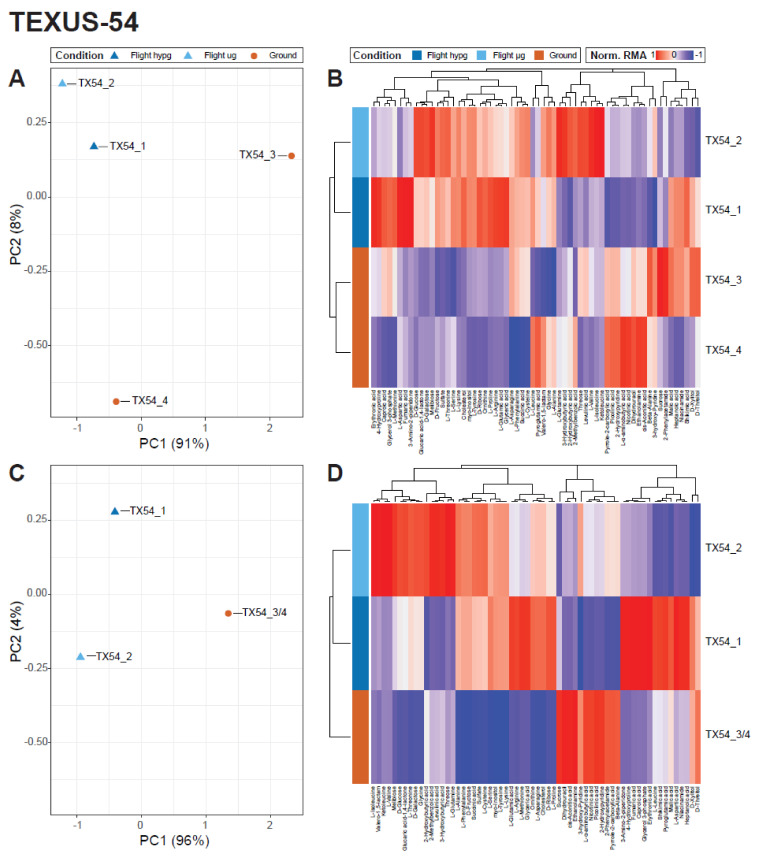
Unsupervised analysis of samples from TEXUS-54. (**A**) Principal component analysis (PCA) plot of the four samples of the mission. The dominant principal component 1 separated ground from flight samples, though the variation along the ground samples was dominant. (**B**) A clustering heatmap plot of the same samples. Normalized relative metabolic abundance (Norm. RMA) is displayed for every metabolite (horizontal axis) for every sample (vertical axis). Cluster grids are proportional to relative differences along samples/groups. Ground and flight samples cluster together which speaks in favor of an effect-driven separation, though the differences within groups are rather large. (**C**) A PCA plot where the two ground samples were merged into a shared ground sample to visualize the average effect of ground samples are combined into one sample group. A clear separation of flight effects can be identified, in which the microgravity sample displays a stronger separation than the hypg sample, which is in line with the microgravity sample being exposed to altered gravity for a longer period of time. (**D**) The clustering heatmap displaying the dataset where the two ground controls were combined.

To assess the datasets for general effects of altered gravity, a principal component analysis (PCA) and a clustering heatmap analysis were performed for both datasets. For TEXUS-54, the four samples showed a clear separation along principal component (PC) 1 which corresponds to the highest variance in the dataset (Figure 4A). The two ground samples (TX54_3 & TX54_4) were exposed to identical conditions, differing only by five minutes of incubation time representing the hypg and µg fixation timepoints. They showed a pronounced separation in the PCA plot and differ more than the two flight samples (hypg: TX54_1, µg: TX54_2), but still clustered in the clustering heatmap plot (Figure 4B). For a better visualization, the two samples were averaged and PCA and heatmap analysis repeated (Figure 4C,D). The averaged ground sample was clearly separated from the two altered gravity conditions in the PCA and the clustering heatmap.

**Figure 5:**
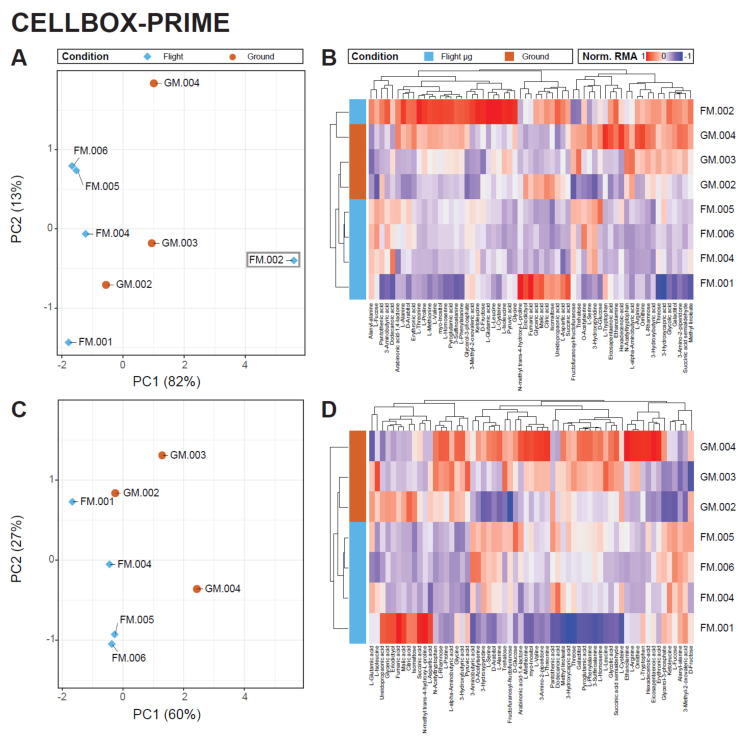
Unsupervised analysis of samples from CELLBOX-PRIME. (**A**) PCA plot of all 5 flight datapoints (in blue) and all 3 ground datapoints (in brown). Principal component 1 represents 82% of total variance in the dataset. Flight samples all localize left of ground samples, except for FM.002. (**B**) Clustering heatmap of the same data. FM.002 does not cluster with any sample from the flight set and has the highest normalized RMA for almost all quantifiable metabolites. Based on A and B and the appearance of the sample chamber indicating potential tightness issues, FM.002 was considered a statistical outlier sample and excluded from further analysis. (**C**) PCA of all samples, excluding FM.002. A distinct clustering of flight and of ground samples along PC1 can be observed. (**D**) Clustering heatmap of the dataset, excluding FM.002. GM.004 and FM.001 are separated from the rest of the samples but do not show RMA value distributions that do not compare to other samples as extremely as FM.002 does.

For the eight CELLBOX-PRIME samples, both the PCA (Figure 5A) and the clustering heatmap (Figure 5B) showed a behavior for sample FM.002 that was atypical for both flight (FM.001, FM.002, FM.004-FM.006) and ground (GM.002- GM.004) samples. In the clustering heatmap, the sample showed the highest normalized relative abundance values for almost all metabolites. After sample recovery from the spacecraft, a precipitate had been discovered on the outside of the corresponding cultivation chamber, indicating sample leakage. FM.002 was thus considered an outlier caused by technical fault and omitted from downstream analysis. A re-analysis of the outlier-removed dataset offered only mediocre separation between flight and ground samples along PC 1 (Figure 5C). However, the heatmap revealed complete clustering of ground samples against flight samples (Figure 5D).

Having two independent datasets that stem from platforms with very different flight profiles and differing exposure time to altered gravity, the datasets were first analyzed on their own and later assessed for similarities.

### 2.2. Identification of Pools of Gravity-Sensitive Metabolites

**Figure 6:**
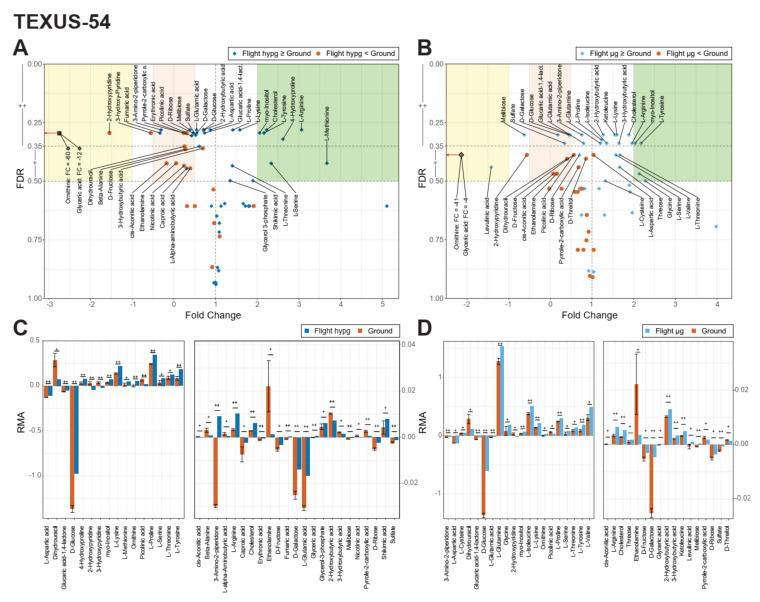
Volcano and bar plots of samples from TEXUS-54. (**A**,**B**) Volcano plots showing the estimated false discovery rate (FDR) against fold change (FC) for the (**A**) comparisons flight hypg/ground and (**B**) flight µg/ground. Because some metabolites showed negative abundance values relative to medium blanks, FC can be negative. The direction of change is indicated by color and shape of the point. As an orientation aid, areas of significant large effect increase (double or more) are highlighted by a light green frame, areas of significant effect decrease (half or less, and inverted if below 0) are highlighted in red, and areas of significant effect inversion (consumption of metabolite to production or production to consumption) with equal or increased effect strength are highlighted in light yellow. (**A**) 41% of all metabolites showed an FDR below 0.35, 63% below 0.5. Ornithine, and glyceric acid showed exceptionally low FCs of −60, and −12, respectively, which exceed the plot margins and are therefore set to smaller values with the correct fold change indicated in their labels. (**B**) 29% of all metabolites showed an FDR below 0.35, 60% below 0.5. Also, Ornithine and Glyceric acid showed exceptionally low FCs of −41, and −4, respectively. (**C**,**D**): Bar plots showing relative metabolite abundance (RMA) for flight hypg (**C**) and flight µg (**D**) and the ground samples for metabolites with an FDR ≤ 0.5. Ground samples are pooled from two biological samples. Error bars represent mean ± standard deviation. +: FDR ≤ 0.5; ++: FDR ≤ 0.35. The plots are split into two subplots with two different linear axes to be able to display all metabolites on linear scales despite their RMA distribution over several orders of magnitude.

**Figure 7:**
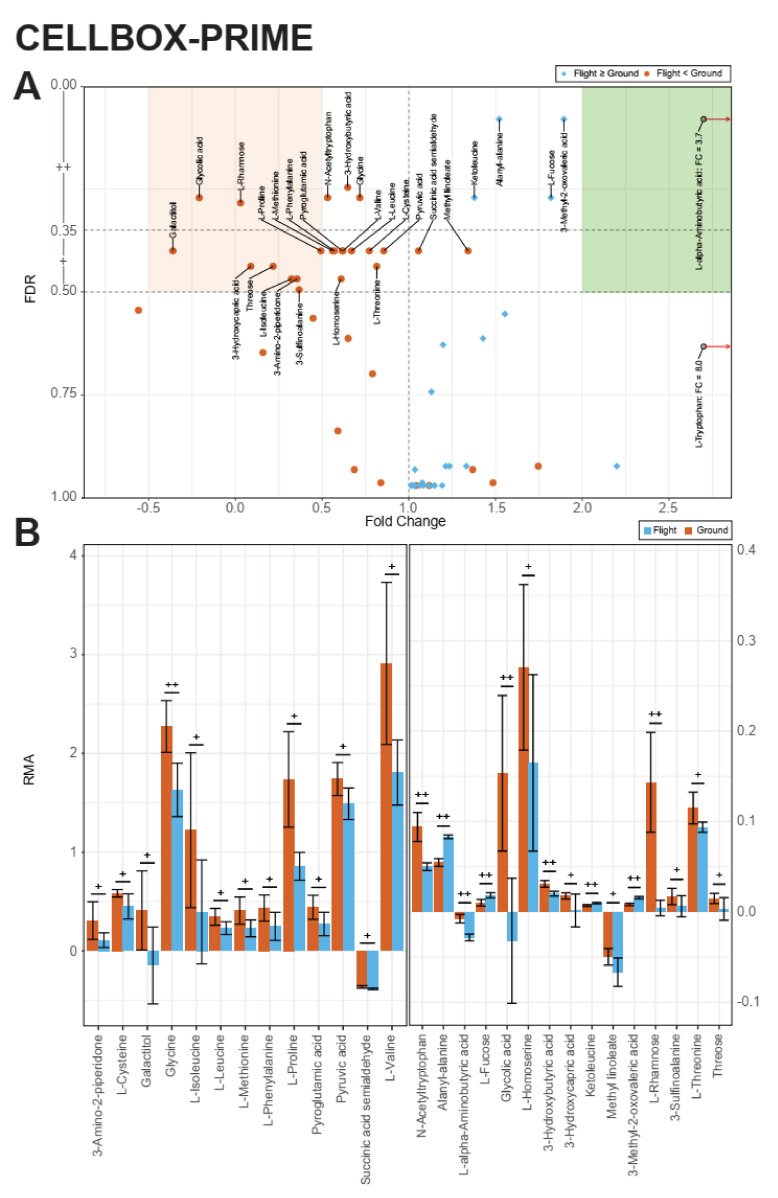
Volcano and bar plots of samples from CELLBOX-PRIME. As an orientation help, areas of significant effect decrease (half or less) are highlighted in red. (**A**) Volcano plot showing the estimated false discover rate (FDR) against fold change (FC) for the comparison flight/ground. The direction of change is indicated by the color and shape of the point. As an orientation aid, areas of significant large effect increase (double or more) are highlighted by a light green frame, and areas of significant effect decrease (half or less, and inverted if below 0) are highlighted in red. It is revealed that 15% of all metabolites showed an FDR below 0.35, 43% below 0.5. L-alpha-Aminobutyric acid and L-Tryptophan both have fold changes that exceed the plot margins, therefore they were set to a fold change of 2.7, and the correct fold change was included in their label. (**B**) Bar plot showing mean ± standard deviation of relative metabolite abundance (RMA) from three ground samples and four flight samples. Only metabolites with FDR ≤ 0.5 are shown. +: FDR ≤ 0.5; ++: FDR ≤ 0.35. The plots are split into two subplots with two different linear axes to be able to display all metabolites on linear scales despite their RMA distribution over several orders of magnitude.

As a next step, the different conditions were assessed for statistically significantly altered metabolites (Figure 6A–D and Figure 7A,B).

**Figure 8:**
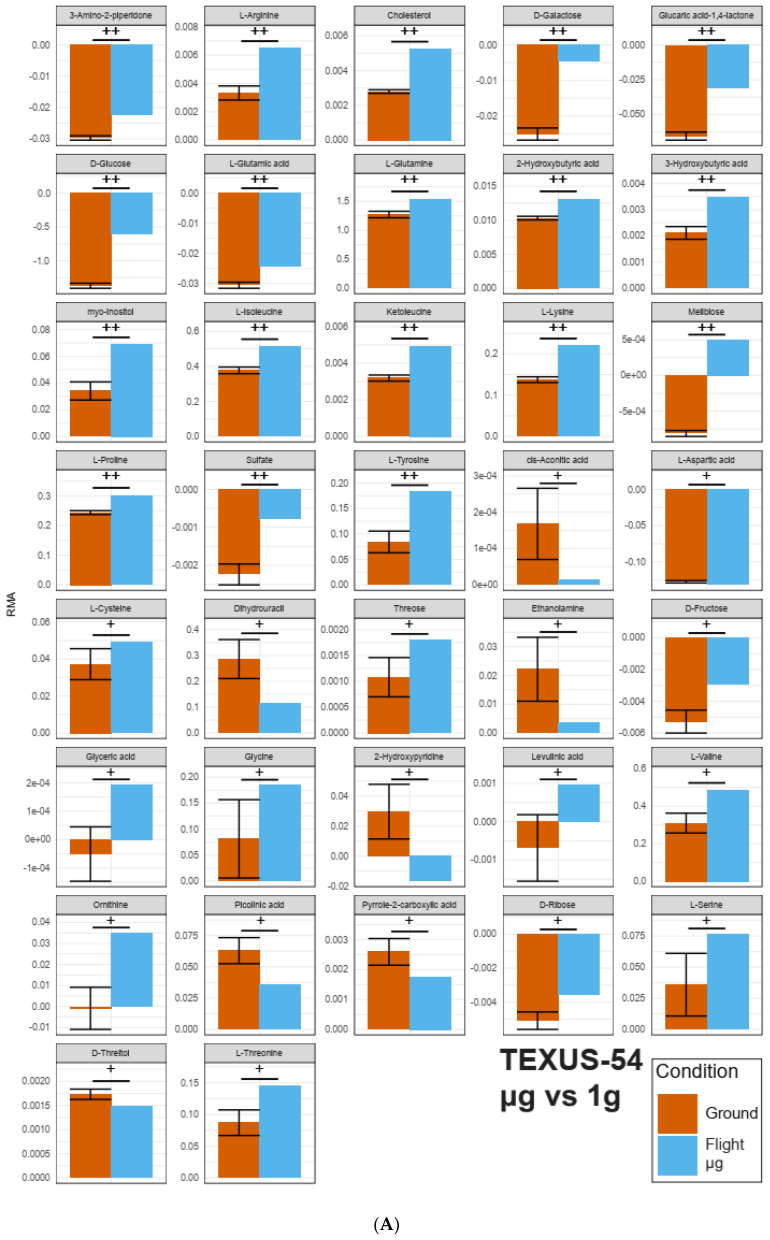

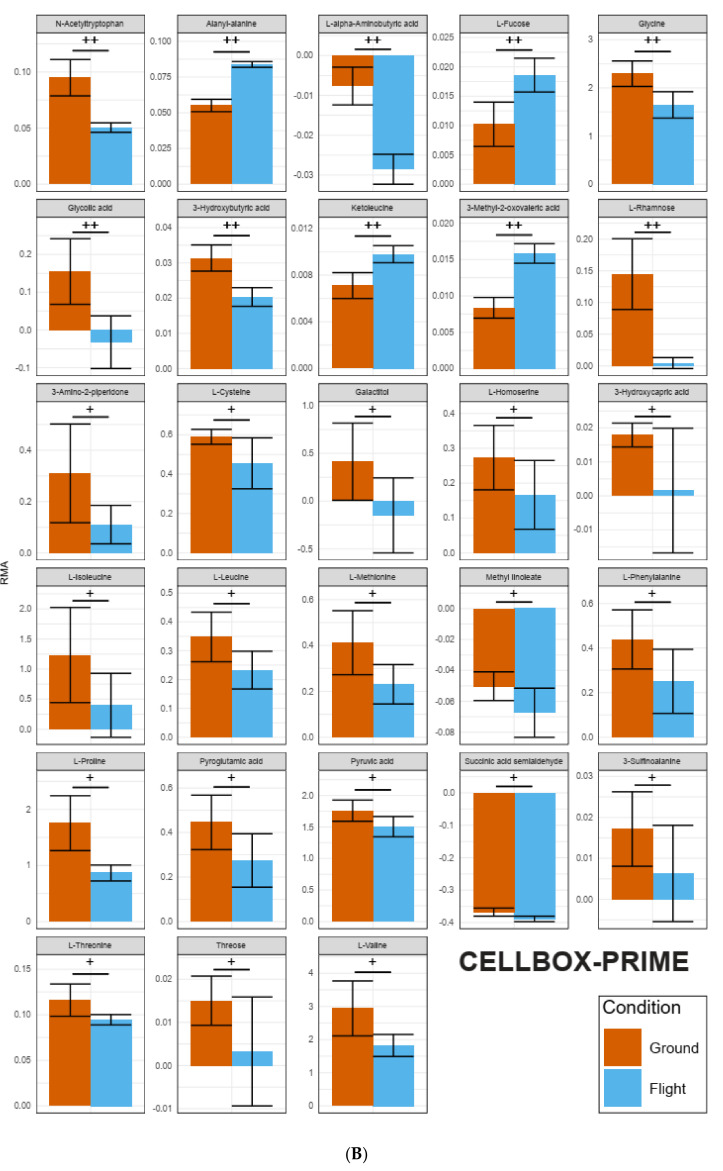
(**A**). Faceted bar plots for TEXUS-54 µg vs. ground control. Only metabolites with FDR ≤ 0.5 are shown. Each metabolite is shown with an individual relative metabolite abundance (RMA) scale. Ground samples are pooled from two biological samples, mean ± standard deviation is shown. +: FDR ≤ 0.5; ++: FDR ≤ 0.35. (**B**). Faceted bar plots for CELLBOX-PRIME. Only metabolites with FDR ≤ 0.5 are shown. Each metabolite is shown with an individual relative metabolite abundance (RMA) scale. Samples are pooled from three (ground) and four (flight) samples, respectively. Mean ± standard deviation is shown. +: FDR ≤ 0.5; ++: FDR ≤ 0.35.

For TEXUS-54, two false discovery rate (FDR) thresholds were defined. A large fraction of metabolites had an FDR of <0.35 (corresponding to raw p values of 0.01–0.13 for hypg against ground and 0.01 to 0.10 for µg against ground, indicated as “++”). A second category was defined for those metabolites with an FDR between 0.35 and 0.50 (corresponding to raw p values of 0.16–0.32 for hypg against ground and of 0.11–0.30 for µg against ground, indicated as “+”). The rationale of including this second tier of metabolites was to include potential candidates of metabolic interaction networks that did not bear sufficient statistical power on their own but could contribute as part of larger clusters. In the given fold change (FC) representation, all metabolites below 0 showed an inverted behavior between the compared conditions (production: RMA above 0 → consumption: RMA below 0, or other way around). We used the term “effect size” to denote the magnitude of either production or consumption of a compound. The hypg against ground comparison (Figure 6A) showed seven metabolites with strong increases in production (fold change (FC) > 2, highlighted in green), namely myo-Inositol, Cholesterol, L-Tyrosine, 4-Hydroxiproline, L-Arginine, L-Methionine, and L-Serine. Additionally, 14 metabolites appeared with strongly diminished effect size or inverted behavior with smaller effect size (−0.5 < FC < 0.5, highlighted in red), such as Beta-Alanine, Dihydrouracil, or Fructose. Finally, the three metabolites Ornithine, Glyceric acid and 2-Hydroxypyridine showed an inverted behavior but with increased effect size (FC < −1, highlighted in yellow). Of these, Ornithine and Glyceric acid had striking fold change values of −60and −12, respectively, which indicated a strong reaction towards hypergravity. To be able to directly compare the abundance values, a bar chart of all significant (“++” or “+”) metabolites was generated, showing the average RMA values and standard deviations (ground samples only) for the respective flight sample and the ground samples (Figure 6C,D and Figure 8A and Supplementary Figure S1 for plots faceted by metabolite with individual vertical axes). The microgravity against ground comparison showed a relatable but slightly less pronounced effect pattern (Figure 6B). Four metabolites were considered strongly increased, but with reduced magnitudes since all significant FCs were below 2.5 (compared to 3.7 for hypg against ground), and seven metabolites were strongly decreased in effect size. Four metabolites were present with inverted increased effects, with the same two metabolites with exceedingly large negative fold changes with only slightly decreased fold change magnitudes (Ornithine with −41, and Glyceric acid with −4). This indicated strong general sensitivity to gravity of these two metabolites. The corresponding bar plot again showed similar patterns (Figure 6D).

Glucose as a key component of the cellular energy household showed decreased consumption compared to ground conditions both in hypergravity and microgravity with a more pronounced effect in microgravity. Since the samples resembled supernatant metabolomics, this finding corresponded to an increased glucose level in the media. This indicated that cells released glucose under conditions of altered gravity, since there was no other potential source of altered glucose levels present in the system.

The CELLBOX-PRIME dataset underwent the same analysis (Figure 7A,B). Analogously to TEXUS-54, two significance bands were defined based on FDR, one below 0.35 (corresponding to raw *p* values of <0.01–0.04) and one between 0.50 and 0.35 (corresponding to raw *p* values of 0.08–0.21) (Figure 7A). Generally speaking, the fold changes of this long-term microgravity dataset were not as pronounced as for short-term microgravity in the TEXUS-54 dataset. Nine metabolites that fell into the two significance bands had a fold change between −0.5 and 0.5, and showed a strong decrease in supernatant concentration in microgravity (red). 3-Methyl-2-oxovaleric acid and L-alpha-Aminobutyric acid showed a roughly two-fold increase in production and a four-fold increase in consumption under flight conditions. The corresponding area above FC = 2 is marked in green. Rhamnose showed the strongest decrease in signal by more than 95%, which was also represented by the large absolute difference apparent in the RMA value bar chart (Figure 7B and Figure 8B for a plot faceted by metabolites with individual vertical axes).

### 2.3. Intra-Experiment Comparison Revealed Potential Gravity-Sensitive Metabolic Networks

Most metabolic processes are tightly coupled to complex upstream, downstream, and parallel metabolic reactions. Therefore, it seemed unlikely that single metabolites should be exclusively gravisensitive. Rather, a cluster of metabolites would be expected to react to altered gravity in a coordinated fashion. To gain a first idea of which clusters to expect from our cellular systems, an intra-experiment correlation analysis was performed. Hereby, the relative metabolic abundances of every metabolite were compared to each other without considering the gravitational conditions by calculating the inter-metabolite Pearson correlation coefficient over all samples per dataset. By this methodology, metabolic clusters should emerge that are coupled to each other by biochemical reactions in our cells. If one metabolite altered in concentration, not only this metabolite but the entire network of coupled metabolites should show a coherent reaction, either increasing or decreasing in concentration, based on the type of biochemical interaction. After identifying such clusters that are present in both datasets, the networks without a significantly profound reaction under altered gravity could be filtered out to leave only such networks that respond to altered gravity.

**Figure 9:**
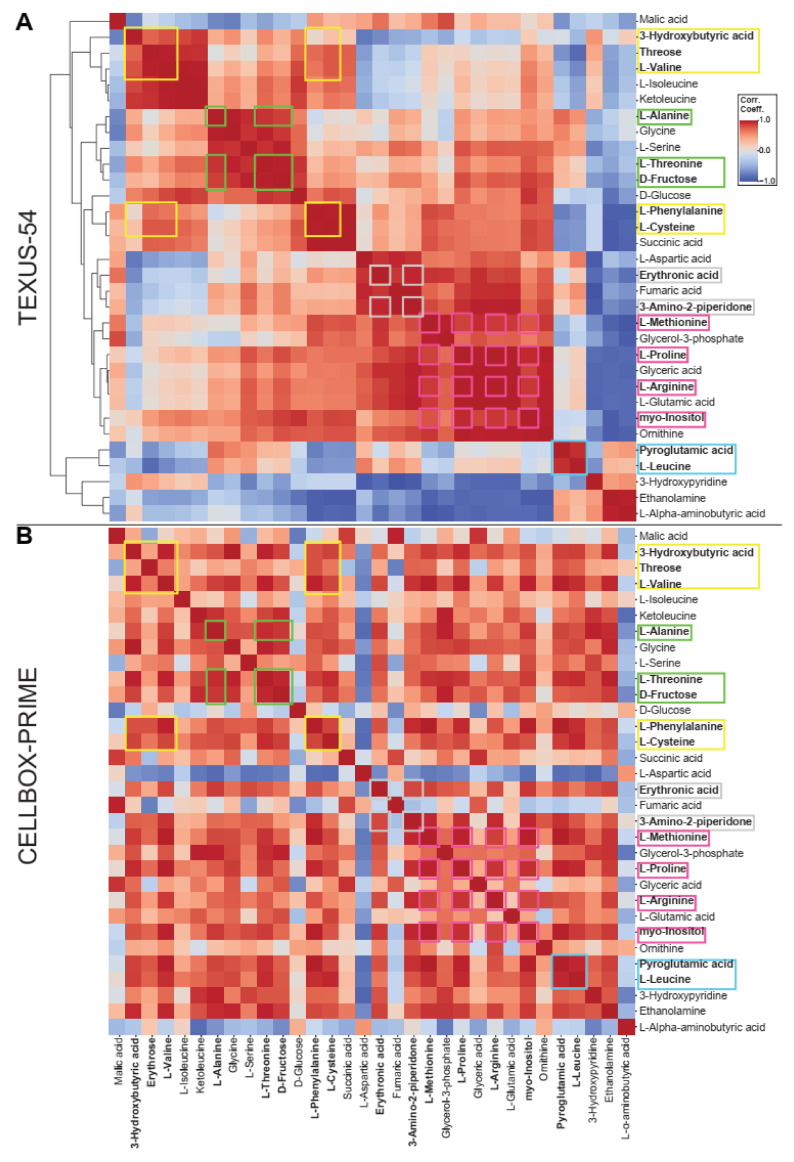
Clustered correlation plot between metabolites for each experiment. Based on the per-sample abundance values, the Pearson correlation coefficients between each metabolite was calculated per experiment. Metabolites that correlate strongly with each other in both experiments are considered a cluster and highlighted with colored frames, one color per group. Clusters are an indicator for metabolic pathways; if elements of clusters are significantly altered, a profound altered gravity effect is indicated. (**A**) Clustered correlation plot for TEXUS-54. Metabolites are ordered by the clustering of their correlation coefficients, which is visualized on the left side of the plot. (**B**) Correlation coefficients for CELLBOX-PRIME. For maximum comparability, the metabolites are in the same order as for A, therefore a mixed pattern emerges that does not follow the clustering for metabolites in CELLBOX-PRIME. A large cluster is present in both experiments centered around Threose and L-Valine and around L-Phenylalanine and L-Cysteine.

To be able to perform such a comparison between the two experiments, a clustering correlation heatmap of metabolites that were present in both datasets was generated for TEXUS-54. Further, an un-clustered correlation heatmap, sorted in the same order as the first heatmap, was generated for CELLBOX-PRIME (Figure 9). The Pearson correlation coefficient ranged from 1 (perfect correlation, an increase in one metabolite leads to an increase in the other) to −1 (perfect anticorrelation, an increase in one metabolite leads to decrease in the other), with 0 as no correlation. A perfect correlation was present on the diagonal line where metabolites are compared to themselves. If a cluster of strong correlation or strong anticorrelation emerged for both experiments, a potential gravity-sensitive cluster had been identified. Entire clustered heatmaps for both datasets that additionally contain metabolites that are only present in one experiment are available in Supplementary Figure S2.

For most regions, a chaotic distribution was the result for the unsorted CELLBOX-PRIME heatmap. One larger cluster emerged from the analysis, marked in yellow, comprising 3-hydroxybutyric acid, Threose, L-Valine, L-Phenylalanine and L-Cysteine (which is weakly associated). Four additional small clusters that did not correlate with the first cluster in both datasets were identified: L-Alanine with L-Threonine and D-Fructose were marked in green, L-Methionine with L-Proline, L-Arginine, and myo-Inositol were marked in pink, Erythronic acid with 3-amino-2-Piperidone were marked in grey, and Pyroglutamic acid with L-Leucine were marked in blue.

### 2.4. An Inter-Experiment Gravity Effect Comparison Identified a Large Gravisensitive Cluster

**Figure 10:**
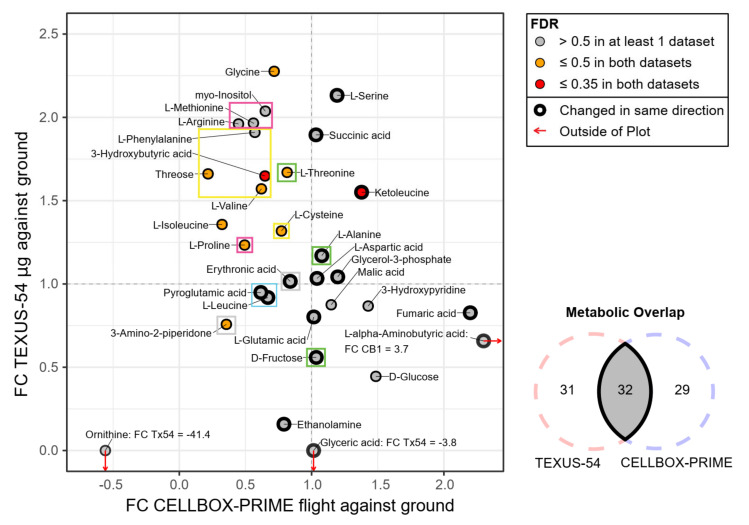
Inter-experiment correlation diagram, plotting the fold changes of shared metabolites. Each dot represents 1 out of 32 shared metabolites. Metabolites that show a difference in the same direction (decrease or increase of RMA value for both sets) are highlighted by a stronger black border. Metabolites that have FDRs lower than certain thresholds are highlighted in yellow or red (comp. legend). Ornithine, Glyceric acid, and L-alpha-Aminobutyric acid all have fold changes that exceed the plot margins; they were set to a fold change of 0 for TEXUS-54, resp. 2.3 for L-alpha-Aminobutyric acid for CELLBOX-PRIME, the correct fold change was included in their label. Clusters that were identified in Figure 9 have been included in the figure by drawing colored boxes around metabolites that are in a cluster group. The plot has four different quadrants, with many metabolites in the top left quadrant with increased concentrations for TEXUS-54 and decreased concentrations for CELLBOX-PRIME, and a center that includes metabolites which do not or only slightly react to altered gravity.

Since the two datasets contained different sets of metabolites that only partly overlapped, showed RMA values within different ranges due to non-similar scaling of the dimension-less RMA values, and showed changes in different directions for certain metabolites, a direct comparison of abundance values was not suitable for a cross-experiment analysis. Out of the 63 metabolites for TEXUS-54 and the 61 metabolites for CELLBOX-PRIME, 32 metabolites overlapped. A two-dimensional projection of the corresponding fold changes of CELLBOX-PRIME flight against ground on the horizontal axis and TEXUS-54 µg against ground on the vertical axis was generated (Figure 10). If the direction of change (less generation = stronger consumption, more generation = less consumption) was the same for a metabolite for both sets, this was indicated by a thick black border, a metabolite that fell into any of the two defined significance bands in both sets was indicated by a yellow or red fill color (comp. legend), and the clusters identified in Figure 9 were indicated by colored boxes. The plot could be divided into four quadrants.

The upper left showed an effect strength increase (stronger consumption or generation) for TEXUS-54 but a decrease for CELLBOX-PRIME. The upper right quadrant had increased signal for both experiments. The lower left implied a decrease for both experiments. The lower right showed a decrease for TEXUS-54 but an increase for CELLBOX-PRIME. Finally, the center contained metabolites that did not show strong effects for both experiments. Metabolites with a fold change below zero were an exception to the above quadrant definitions since their direction of change is altered.

The metabolic profiles of TEXUS-54 µg and hypg are highly comparable for some metabolites (Supplementary Figure S3). For these metabolites, this indicates a general altered gravity-induced reaction (in contrast to microgravity-specific mechanisms), or that, for these metabolites, the effects for TEXUS-54 already occurred during the hypergravity phase. To be able to differentiate microgravity from hypergravity effects, for each identified cluster the metabolites were identified that were already significantly altered in hypergravity (compare Figure 6A and Table 1).

The large previously identified cluster (indicated in yellow) showed consistent behavior in terms of FC for all metabolites which thus also clustered in the inter-dataset correlation plot. All members showed reduced concentrations during long-term microgravity onboard the ISS but increased concentrations after 5 min of microgravity in the TEXUS-54 rocket with increases between 30% to almost 100%. Additionally, Threose, L-Valine, and L-Cysteine fell into the lower significance band and 3-Hydroxybutyric acid even in the upper significance band in both datasets. Only 3-Hydroxybutyric acid fell into the lower significance band for TEXUS-54 hypg. Therefore, this amino acid-rich cluster is of high interest for gravisensitive metabolic pathways in macrophages.

To assess functional and chemical relationships between those metabolites, amino acids were compared against the sets of essential/nonessential, ketogenic/glucogenic, and neutral/charged amino acids. However, no relationship could be identified. Except for L-Cysteine, all amino acids were categorized as essential amino acids, therefore a shared metabolite-generating process was unlikely to be the reason. Rather, decreased consumption/degradation or an increased output of these amino acids from the cells under altered gravity emerged as possible explanations.

L-Proline, L-Arginine, myo-Inositol, and L-Methionine were localized adjacently to the large cluster but are not part of it. They showed similar regulation with L-Proline falling into the upper significance band; therefore, these four metabolites were further candidates of interest. However, for TEXUS-54 hypg, they all already appeared significantly altered. The green cluster, containing L-Threonine, L-Alanine and D-Fructose, showed strong reactions for the TEXUS-54 comparison but not for the CELLBOX-PRIME-comparison. From these, L-Threonine and D-Fructose were significantly altered in the TEXUS-54 hypergravity comparison. The metabolites from the green cluster did not all fall into significance bands for TEXUS-54 µg and CELLBOX-PRIME, but still showed interesting behavior. Therefore, they were potential candidates for initial gravity-sensitive effects that were brought back to pre-effect levels after some time.

Erythronic acid showed a slight decrease in effect strength under short hypergravity and only showed subtle behavior under short microgravity. 3-amino-2-Piperidone on the other hand did show an inversion under short hypergravity and a medium decrease for both experiments under microgravity. Therefore, the grey cluster was not considered consistently regulated. Even more prominently, the blue-colored cluster showed almost no reaction to short microgravity and hypergravity and only rather subtle decreased reactions towards long-term microgravity. Therefore, L-Leucine and Pyroglutamic acid were part of potential general pathways but were not considered gravity-sensitive. To the right of this cluster, there were L-Aspartic acid, and Glycerol-3-phosphate in the center that virtually did not show any reaction in both experiments and were therefore considered stable under short- and long-term altered gravity.

An imbalance of the distribution of metabolites between the four quadrants was evident. Most metabolites that showed substantially altered concentrations in both experiments were localized in the upper left quadrant. This effect was even more pronounced if only significant metabolites are considered.

Ketoleucine was the only candidate that showed an appreciable and significant concentration increase in both experiments for microgravity. However, no changes were detected in hypergravity. No direct coupling could be detected for the metabolite (Figure 9), but being a product of incomplete ketogenic amino acid degradation, it was likely indirectly coupled with other amino acids.

## 3. Discussion

Cellular reactions towards microgravity occur on a variety of molecular levels on very different timescales. The first effects are observed after only one second of microgravity [9,10] and after 20 s large fractions of the cellular transcriptome pool undergo significant changes that globally affect many different gene loci on all chromosomes without specifically targeting isolated genetic pathways [11,12,13]. Generally, the effects of microgravity are very broad and complex [18]. So far there is still a lack of general hypotheses on underlying mechanisms that holistically rationalize the number of effects. Therefore, there is now a need for advanced studies like correlative studies which have the potential to shed light on underlying mechanisms since they are able to discriminate platform- or cell-specific effects from generally conserved mechanisms of reactions to altered gravity. Metabolomics, as previously described, is a highly sensitive omics discipline with ultrashort minimum trigger reaction times and is likely to be affected by broad changes already after short microgravity exposure [21]. Consequently, metabolomics is particularly suited for studies on the effects of short-term microgravity.

This study comparatively assessed the effects of short-term and long-term microgravity on the supernatant metabolome of primary human macrophages. Two microgravity platforms were compared for this purpose: a short-time suborbital ballistic rocket flight during the TEXUS-54 campaign, providing 50 s of hypergravity and 325 s of microgravity including 300 s of high-quality (10^−5^–10^−6^ g) microgravity [30,31], and the International Space Station with 158 s hypergravity before main engine cut-off (MECO) and 9 min before 2nd stage engine cutoff (SECO) for the Falcon-9 and 11 days of microgravity (quality of 10^−3^ g) during the CELLBOX-PRIME experiment.

On both platforms, altered gravity induced profound alterations of the supernatant metabolome. Out of ~60 detected metabolites per platform, 18 fell in the higher differential significance band (FDR ≤ 0.35, Figure 6B and Figure 7A) for short microgravity and 10 fell into the higher differential significance band for long-term microgravity. From these 18 highly significant metabolites under short microgravity, 14 already appeared to be significant under hypergravity (Figure 6A,B and Table 1).

We observed that the fold changes of the long-term microgravity dataset were not as pronounced as in the short-term altered gravity dataset. This might indicate a re-adaptation after the initial trigger caused by changes of the gravity environment. Similar re-adaptation behavior was described for human U937 and Jurkat immune cell lines when comparing transcriptional effects of 20 s of microgravity on parabolic flights to 300 s of microgravity on suborbital ballistic rockets: genes that were differentially expressed on the parabolic flight were no longer differentially expressed on the rocket platform, but a pool of new differentially expressed genes emerged [11,13]. We were further able to demonstrate in the fold change correlation analysis that most significantly altered shared metabolites even showed increased effect strengths during short-term microgravity but then effect strengths even below ground levels during long-term microgravity. This study is another piece of evidence that conserved mechanisms of strong rapid response and consecutive readaptation may be an important cellular principle to quickly cope with the effects of altered gravitational environments on multiple omics levels. This would also give a hint on the “gravity-paradoxon”, that biological systems drastically react to short phases of microgravity, but humans are able to withstand microgravity conditions for many months without severe impacts [26,32]. In this context, it is possible that highly dynamic rapid effects induced by the gravity environment lead to coordinated “profile preserving” cellular changes in a non-equilibrated cellular state [33].

A large number of shared metabolites in the quadrant that showed over-re-adaptation behavior (top left quadrant in Figure 10) was identified to belong to the same two intra-dataset metabolite clusters (yellow and pink in Figure 9 and Figure 10). The largest cluster, comprising Threose, L-Valine, L-Phenylalanine, L-Cysteine and, as a weakly-coupled member, 3-Hydroxybutyric acid, consisted exclusively of metabolites showing over-re-adaptation behavior and its metabolites showed comparable fold change values. Except for L-Phenylalanine, this cluster exclusively contained metabolites that fall into the first or second significance band in both datasets. From these identified metabolites, only 3-Hydroxybutyric acid showed already significant fold changes during the TEXUS-54 hypergravity phase (Figure 6A, Table 1). This might indicate a relation of the metabolites as a gravity-sensitive amino acid-related cluster that exclusively reacts to microgravity but not hypergravity. The cluster around L-Arginine, indicated in magenta, is not coupled to this cluster and is less significant, but shows similar behavior. However, most of its metabolites are already significantly altered during hypergravity (Table 1). It therefore might be an independent altered gravity-sensitive cluster. Generally, the parallel involvement of multiple amino acids in these clusters could be due to the tight interlinkage of amino acids due to several pathways [34].

Given that the data reflect media concentrations, the question arises why cells secrete large amounts of amino acids into the media and take it up on a longer timescale. This could be a general stress response of the cells due to microgravity or general altered gravity conditions. Considering the microgravity-specific findings for Ketoleucine and that it (anti)-correlates with the large amino acid cluster gives a hint that the amino acid homeostasis may be affected [35]. It is well established that immune cells use increased amino acid catabolism and thereby altered amino acid levels as activators of immune response pathways, including inflammatory responses [36,37,38,39]. This interaction is promoted via mTOR and GCB2 pathways since these sense intracellular amino acid levels and convert them into cellular responses via GCN2- and AhR-signaling [40]. Observed immunological responses to altered gravity could therefore (partly) occur due to altered amino acid levels inside cells. Interestingly, amino acids have also been reported to be among the most significantly altered metabolites in the NASA twin study [26].

It remains speculative if these clusters formed due to a shared gravity-sensing mechanism that directly acts on the abundance of these specific amino acids or not. This could be due to a shared gravity-sensitive process that affects the internal concentration of amino acids by either forming or degrading them, or due to a common gravity-sensitive export mechanism [41] (enhanced secretion of these amino acids after short microgravity, enhanced re-import after prolonged phases of microgravity). Generally, gravitational effects on biochemical processes that lead to altered metabolite concentrations can occur either indirectly, due to regulatory pathways affecting enzymes, or directly, due to altered diffusion behavior (absence of Raleigh convection), absence of hydrostatic pressure with implications on reaction equilibria, altered membrane diffusion of protein complexes, and changes in enzyme/enzyme complex conformation [30].

Pinpointing the source of the increased amino acid concentrations after short microgravity would be a promising way to predict the underlying mechanism. A shared formation mechanism is yet rather unrealistic due to the high number of contained essential amino acids (L-Phenylalanine, L-Valine, L-Methionine, and L-Arginine as semi-essential amino acids) that are not generated inside the cells [42]. Further, the affected amino acids could not be categorized as being all polar/non-polar or ketogenic/glucogenic. Interestingly, the amino acids in the magenta (Figure 10) group cluster together with Glycine (highly altered), Cysteine and Aspartic acid, which are abundant in the N-/C-terminal degron signals that occur in human cells [43,44,45,46]. These short terminal amino acid sequences are usually part of unordered polypeptide chains that govern protein degradation [47]. As terminal members of polypeptide chains, forming a thermally unstable end of a protein, they are the first amino acids to get cleaved off upon degradation, either N- or C-terminally [48]. Since one central cellular reaction to environmental stress is ubiquitin-related protein degradation [49], the appearance of all degron-relevant amino acids in the highly increased magenta (Figure 10) cluster or adjacent to it (except for Aspartic acid) suggests starting protein degradation as a stress response as the most likely source of increased amino acid levels after short-term microgravity. The degron pathway of the amino acid with the strongest increase in the TEXUS-54 dataset, glycine, is targeted by Kelch family substrate adapters [44,45]. One prominent member, KLHDC1, recognizes C-terminal -GG degrons [50]. This mechanism is especially interesting since the protein is significantly overexpressed in different immune cell lines after 75 s of hypergravity on TEXUS rockets (expression datasets GSE101309 and GSE101102 from [11,12]).

In contrast to the aforementioned metabolites, there are a few that hardly show any reaction to hypergravity and microgravity exposure. L-Aspartic acid as one of the most central candidates shows an insignificant increase by a few percent under microgravity and a non-significant decrease under hypergravity (Table 1). Considering the degree of interconnectivity between amino acid metabolism and central metabolites in general [34], it appears unlikely that L-Aspartic acid is not connected to any pathway which reacts to microgravity or hypergravity. It appears more likely that this is due to a tight regulation of L-Aspartic acid levels in cell supernatants. If verified by orthogonal experiments on different platforms, L-Aspartic acid and other gravity-stable metabolites could be used as normalization/reference targets, as published for transcripts previously [13,51].

Ornithine, a degradation product of proteinogenic amino acids, was present in both datasets. In the TEXUS-54 microgravity and hypergravity datasets, it was significantly upregulated (category “+”, Table 1). In the long-term dataset CELLBOX-PRIME, it appeared strongly downregulated but not in the top significance category. Therefore, the uncommonly strong fold change of Ornithine from little net consumption at 1 g to high net production in microgravity and hypergravity for TEXUS-54 and the opposite effect for CELLBOX-PRIME makes it a potentially interesting target. Ornithine is mainly generated by hydrolysis of arginine via arginase [52]. In the cellular context, Ornithine is then further degraded into putrescine by Ornithine decarboxylase [53], a polyamine. These highly regulatory molecules are involved in a myriad of cellular regulations, including transcriptional regulation via DNA structural changes [54,55], cell proliferation [56,57], scavenging of ROS inside the cell [58,59,60], binding to membrane proteins [61,62], and regulating stress response protein expression ([63,64,65], reviewed in [66]). Since many of these cellular functions have been demonstrated to be affected by microgravity, the potentially highly gravity-sensitive Ornithine and the associated polyamine-dependent regulatory functions are an interesting target for future studies.

Upon comparing single shared metabolites between both datasets, Ketoleucine was identified as the only metabolite that is significantly increased under microgravity conditions in both experiments (Figure 10). Moreover, in contrast to Ornithine, it did not show any reaction under hypergravity (Table 1). No readaptation reaction could be observed for this metabolite. It does not consistently couple to other amino acids/metabolites in general in both experiments; it just shows an association with the large amino acid cluster in the TEXUS-54 dataset and a certain anticorrelation with it in the CELLBOX-PRIME dataset. Therefore, it is a metabolite that could probably serve as a general indicator of microgravity exposure for short and long microgravity based on supernatant analysis, a metabolic microgravity-exclusive marker. Ketoleucine (=alpha-ketoisocaproic acid) is mostly produced due to incomplete degradation of branched-chain amino acids. Such an increased amino acid degradation with elevated levels of Ketoleucine has been shown for C2C12 muscle cells in unloading conditions [67], a state that is used to mimic conditions of spaceflight [68]. Ketoleucine is present in the human body at low levels with muscle tissue as the main production site and is degraded in the liver [69]. It can be converted into Leucine by transamination and has been discussed as a dietary supplement in uremia patients [70,71]. Elevated levels of Ketoleucine do not generally occur in healthy individuals. However, in certain diseases, including maple syrup disease, elevated levels have been reported which have been associated with neurotoxic and metabotoxic behavior [72]. Diseases associated with elevated Ketoleucine levels commonly involve a malfunction of the main degradation route via the branched-chain α-keto acid dehydrogenase complex [73]. This enzyme complex with its ~24 subunits and a total weight of 4–5 MDa is among the largest complexes in human cells and spans the mitochondrial membrane [74,75]. It has been shown that membrane proteins could experience pronounced functional impairments in microgravity due to changing membrane fluidity [76]. This effect could probably explain the increase of Ketoleucine in microgravity: the disturbed amino acid degradation under such conditions could be related to inhibited Ketoleucine dehydrogenation using the branched-chain α-keto acid dehydrogenase resulting from changed membrane fluidity, leading to accumulation of Ketoleucine. Further, since in the physiological environment of the body, one main degradation route of Ketoleucine is the liver [69], the in vitro system used in this study will further lack Ketoleucine degradation capacity. The cellular media replaces the in vivo blood environment, therefore the released Ketoleucine would be transported to the liver in in vivo conditions. The potentially reduced intracellular degradation capacity of ketoleucine and missing degradation in the liver renders Ketoleucine a quasi-stable metabolic endpoint that can only accumulate. With this unique significant accumulation behavior, Ketoleucine could act as an indicator of mid- and long-term altered gravity, not only for the cell via branched-chain amino acid signaling [77], but also for future metabolomics studies in altered gravity. Interestingly, increased Ketoleucine concentrations were detected in the cerebrospinal fluid (CSF) with ageing [78], while metabolic changes that typically accompany aging have been consistently observed in space [79].

In our study, we observed that microgravity exposure leads to an increased abundance of amino acids in the cell culture supernatant of primary human macrophages (Figure 10). A possible explanation could be the microgravity induced modification of protein metabolism and associated degradation of proteins leading to an increased level of amino acids. A modified protein metabolism in microgravity, especially in the form of loss of muscle mass, is a well-known phenomenon and has been described in humans as well as in model organisms such as mice [80,81,82,83].

Additionally, it has been shown in spaceflight and bed rest studies that protein synthesis activity declines upon exposure to real or simulated microgravity conditions requiring an increased dietary protein intake [84,85,86]. During our TEXUS-54 experiment, we identified increased levels of amino acids and glucose in microgravity compared to ground control samples. However, during our CELLBOX-PRIME experiment we observed a reversed situation, i.e., decreased amino acid and glucose levels in the cell culture supernatant. This could be explained by a microgravity-induced reduction of protein synthesis and an increased protein degradation in favor of the production of glucose via gluconeogenesis during short-term microgravity. While after 5 min of microgravity, levels of amino acids and glucose are increased, a reduction is observed after 11 days of microgravity. This is an indication for a later increased consumption. Ketogenic amino acids like Leucine cannot be converted to glucose, but are degraded in the amino acid catabolism by transamination to Ketoleucine [34]. Protein degradation usually occurs as a stress response, for example in starvation conditions or during oxidative stress, for example when reactive oxygen species (ROS) damage proteins [87]. We could show in previous studies that ROS production is highly gravity-dependent and reacts to gravitational stimuli within seconds [9,88]. Thus, ROS metabolism might contribute to the regulation of protein degradation in microgravity.

In this study, we could demonstrate the fundamental effects of altered gravity on the supernatant metabolite composition of human macrophage cells. We postulated that altered protein degradation could represent the underlying reason of the observed metabolic changes. This could either be the consequence of a general effect like cellular autophagy, an important mechanism in inflammatory response and phagocytosis for macrophages [89], a specific response to a different cellular environment [90] or regulated by the macrophageal proteasome system [91], which has been identified as sensitive to microgravity during in vivo experiments on board Space Shuttle mission STS-90 [92]. The proteasome system responds rapidly to oxidative stress [93] and could therefore represent an integrative system for different primary and secondary effects of altered gravity. To understand the metabolic homeostasis could therefore shed light on the potential underlying molecular mechanisms of functional deterioration of human macrophages in microgravity, including reduced phagocytic index, reduced engulfing, oxidative bursting and degranulation [94,95], as reviewed in [96]. Integrative omics analysis has the potential to pinpoint certain metabolic mechanisms: intriguingly, Shi et al. reported the urea cycle to be among the transcriptional significantly affected pathways in macrophages after 12 days in microgravity, potentially compensating for the increased Ornithine concentrations after five minutes [97].

Nevertheless, further studies are required to better understand the gravity-sensitive nature of these amino acid clusters, their stimulability by hypergravity versus microgravity, their relations with protein degradation and with Ketoleucine. A deeper understanding would potentially allow for prediction of a functional mechanism. Further, multi-omics studies could elucidate if the lower concentrations after prolonged exposure are related to differential expression of a membrane-spanning transporter or other amino acid metabolism-associated enzymes.

In summary, this comparative study was able to demonstrate the pivotal effects of both short and long-term microgravity and partly short-term hypergravity on the metabolite concentrations in human primary macrophage cell culture supernatants and detected an adaptation reaction for cellular metabolites in microgravity. Additionally, a gravity-sensitive metabolite cluster that extends across experimental platform borders could be postulated, potentially associated with amino acid degradation. Further correlative studies might shed light on the conservation status of such an amino acid cluster and the potential underlying mechanisms.

## 4. Materials and Methods

### 4.1. CELLBOX-PRIME/SpaceX CRS3

The CELLBOX-PRIME experiment is described briefly; a detailed description of the experiment can be found in Tauber et al. [17].

#### 4.1.1. Preparation of Primary Human Macrophages

Primary human macrophages were differentiated from peripheral blood monocytic cells by PromoCell (Heidelberg, Germany). They were cultured adherent on polycarbonate slides that fit into the flight hardware. Prior to experiment integration they were transported by airplane to the Space Life Science Labs (Exploration Park, Kennedy Space Center, FL, USA).

#### 4.1.2. Hardware Concept

The experiment was designed to expose cells to different gravity conditions and subsequently retrieve cell culture supernatant for analysis by metabolomics and fixed cells for analysis by immunocytochemical staining. This was made possible by the hardware Biorack type I standard CELLBOX-PRIME EUE type IV container from Airbus Defense and Space. Cells were rinsed with fixation fluid (2% paraformaldehyde and 1.3% sucrose). At the same time the cell culture supernatant was pushed from the culture chamber into a hardware compartment where it could be sampled later. Fixed cells were rinsed with PBS to avoid overfixation. One experimental unit held four slides with adherent cells (see Figure 2 in Tauber et al. [17]).

#### 4.1.3. Experiment Integration and Upload

Six days before launch, the primary human macrophages were integrated into Biorack type I standard CELLBOX-PRIME EUE type IV containers. All hardware components were sterilized by autoclaving or ethylene oxide exposure and integration was done under sterile conditions. Six experimental containers (units) were prepared for the ISS, and three were prepared as 1 g ground references, staying in the laboratory on the ground. Two days before launch, the medium in the experimental units was changed to resupply the cells with fresh nutrients. The units were kept at 37 °C from integration until loading into the spaceship. From then on until unloading on the ISS they were kept passively between 20 and 37 °C. The experiment was uploaded with the SpaceX CRS-3 mission on April 18, 2014 from Cape Canaveral SLC-40, Florida. On the ISS, units were mounted into the “NanoRacks Astrium Centrifuge” (OpNom: NanoRacks BioRack Centrifuge) in the U.S. National Lab.

#### 4.1.4. Experiment Design and Sampling

Six experiment containers were uploaded to the ISS. After 11 days, 5 units were fixed on the ISS and consecutively stabilized. One container did not release and therefore remained unfixed; it was consequently excluded from further metabolic analysis. The three ground control units were treated in parallel on the same day (see Figure 2). The units were kept at 4 °C after fixation and downloaded with the Dragon spaceship on 18 May 2014. After retrieval of the samples in the Space Life Science Labs (Exploration Park, Kennedy Space Center, FL, USA), samples were transported to the University of Zurich for analysis. A scheme of all resulting sample groups can be seen in Figure 2.

### 4.2. TEXUS-54

#### 4.2.1. Isolation and Cryopreservation of Human Monocytes

Peripheral blood monocytic cells (PBMCs) were isolated from buffy coats which were obtained from a blood transfusion service in Zurich. Donors had given their written consent that samples might be used for research. All steps were done at room temperature if not stated differently. The buffy coats were diluted 2:1 with PBS (Biochrom GmbH, Berlin, Germany) and 15 mL were layered on 10 mL Ficoll Paque Premium (GE Healthcare Bio-Sciences, Uppsala, Sweden). After centrifugation for 30 min at 400× *g*, the layer of PBMCs was collected. Cells were washed 4 times with 50 mL PBS. During the first wash, centrifugation was 10 min at 400× *g*, and in the following 3 washes centrifugation was 10 min at 350× *g*. To isolate monocytes from the PBMCs, cells from one buffy coat were resuspended in 20 mL of Mononuclear Cell Medium (Promocell, Heidelgerg, Germany) and layered on 25 mL of a 46% Percoll solution (10.64 mL Percoll (GE Healthcare Bio-Sciences, Uppsala, Sweden), 13.5 mL RPMI (Biochrom GmbH, Berlin, Germany), 0.86 mL 10× PBS (Sigma-Aldrich Chemie GmbH, Steinheim, Germany)). After centrifugation at 550× *g* for 30 min, monocytes were collected from the interphase, washed 1 or 2 times in 50 mL PBS until the supernatant was clear, and resuspended in Mononuclear Cell Medium (Promocell, Heidelberg, Germany). After quantification, cells were resuspended in serum-free Freezing Medium (Promocell, Heidelberg, Germany) at a concentration of 15 Mio/mL. Then, 1.5 mL aliquots (22.5 Mio cells) were frozen at −80 °C and transferred to −150 °C within 24 h for storage.

#### 4.2.2. Preparation of Primary Human Macrophages from Cryopreserved Human Monocytes

Cryopreserved monocytes were transported from the University of Zurich in Switzerland to the ESRANGE Space Center laboratories in Sweden at −80 °C, 23 days before the experimental flight. Monocytes were differentiated into primary macrophages using M1-Macrophage Generation Medium DXF by PromoCell (Heidelberg, Germany) and the related protocol for differentiation of M1-polarized Macrophages with modifications related to cryopreserved cells as starting material. Eleven days before the experimental flight, cells were thawed by slewing the cryovials in a 37 °C water bath for 2 min. Subsequently, the cell-suspension was added to 20 mL of MCM (PromoCell, Heidelberg, Germany) (equilibrated for 20 min in a 37 °C CO2 incubator) and incubated in a cell culture incubator (37 °C, 5% CO2, 95% humidity) for 11.5–13 h. Subsequently, cells were pelleted by centrifugation at 350× *g* for 10 min and resuspended in M1 Macrophage Generation Medium DXF to a concentration of 3,4 Mio/mL for differentiation. Then, 1.5 mL of this suspension was pipetted onto dry cell-support surfaces of the flight hardware which had been placed in 4-well plates (Sarstedt, Nümbrecht, Germany) beforehand to let cells attach. The cell suspension was spread over the whole surface to achieve a homogeneous distribution of adherent cells. Rectangular cell-support surfaces and H-shaped cell-support surfaces were used (see hardware concept). After 30 min–2 h, cells had attached to the surface and 7.5 mL of M1 Macrophage Generation Medium DXF was added to the wells of the rectangular type cell-support surfaces. In contrast, on the H-shaped cell-support surfaces, cells were seeded on both sides. Therefore, surfaces were flipped over, and medium was pipetted into the space between the 4-well plate and the cell-support surfaces to prevent the attached cells from drying out. Then, 1.5 mL of freshly prepared cell solution (3.4 Mio/mL in M1 Macrophage Generation Medium DXF) was pipetted onto the side which was now on top and cells could attach for 30 min to 2 h. Subsequently, 7.5 mL of M1 Macrophage Generation Medium DXF was added to the wells. Cells on both types of cell-support surfaces were incubated for 6 days to allow differentiation. Half of the cell culture supernatant (4.5 mL) was removed and 4 mL of fresh medium was added to the wells. Cells in the culture supernatant were collected by centrifugation for 10 min at 300× *g*, resuspended in 1 mL of fresh medium and carefully pipetted on top of the cell-support surfaces. Three days later, a compete medium exchange was performed: Medium was removed from the wells, and 8 mL of fresh M1 Macrophage Generation Medium DXF was added to the wells. Cells from the old medium were collected by centrifugation for 10 min at 300× *g*, resuspended in 1 mL of fresh medium and carefully pipetted on top of the cell-support surfaces. Two days later, cell-support surfaces were integrated in the FLUFIX-hardware (cf. next section) and subjected to the experimental flight or left in the cell culture incubator as cell culture control samples, respectively.

#### 4.2.3. Experiment Hardware

The FLUFIX device, a flight hardware solution offered by Airbus DS (Bremen, Germany), was used. It enables the replacement of fluid in cell culture chambers at defined times during sounding rocket flights of the DLR-TEXUS type. Figure 1A depicts a scheme of a FLUFIX-cassette, showing that it comprises two culture chambers next to each other. They are connected to a compartment (syringe) with 50 mL of fluid. Upon opening of a solenoid valve, 48 mL of this fluid flow into and through the culture chambers; the conditioned medium in the culture chambers (9 mL each) and 30 mL of the fluid from the syringe is pushed into a sampling compartment where the samples for the metabolomic analysis were taken after flight (Figure 1A). The conditioned medium from both culture chambers of one cassette is collected in the same sampling compartment, therefore one cassette represents one sample for metabolomic analysis.

To accommodate the adherent primary human macrophages in the culture chambers, cells were seeded onto cell-support surfaces which fit in the culture chambers. The cell-support surfaces were cut out of the bottoms of T175 cell culture vessels. Two cell-support surfaces were fitted within one culture chamber. Each had a rectangular shape of 62 × 20 mm. Cells were seeded on one side of the surface and it was placed in contact to the bottom of the cell culture chamber. The other one had a H-shape with an area of 55 × 25 mm and 4 extensions, allowing the mounting of four O-rings as spacers. Cells were seeded on both sides of this support, and it was placed between the rectangular slide and the lid of the chamber with enough distance to allow fluid flow (Figure 1E–G). With this method, three layers of cells with 5 Mio seeded cells each, 15 Mio in total, were installed in one chamber.

#### 4.2.4. Experiment Design, Conduction, and Sampling

The experiment was designed to assess the effect of 325 s of microgravity on the spectrum of metabolites in the cell culture supernatant of primary human macrophages. It was conducted on TEXUS-54, a suborbital ballistic rocket flight of the German Center for Aerospace (DLR). At defined times during different gravity conditions, culture supernatant was separated from the cells. The fluid injected into the cell culture chamber was a fixative solution, as in a separate analysis [31] the fixed cells were investigated.

Two FLUFIX modules were available, one was implemented in the rocket, the second one remained on the ground holding the ground control samples, called ground-module. Each module holds two cassettes allowing in total for two flight samples and two ground control samples in identical hardware.

The experimental setup comprised the following four samples (comp. Figure 2):

(1) Flight-baseline (BL) sample: cell culture supernatant was separated after the hypergravity phase of the suborbital ballistic flight, directly before the microgravity phase, 50 s after launch.
(2) Flight-microgravity (µg) sample: cell culture supernatant was separated after 325 s of microgravity, 375 s after Launch.
(3) 1 g hardware (H/W) ground control BL-time: samples were in the ground-module and cell culture supernatant was separated concomitantly to the Flight-BL sample.
(4) 1 g hardware (H/W) ground control µg -time: samples were in the ground-module and cell culture supernatant was separated concomitantly to the Flight-µg sample.

Cells of samples (1)–(4) were integrated into the FLUFIX culture chamber 5.5 h prior to launch on 13 May 2018. The two cell culture chambers of one cassette were filled with primary human macrophages of two different donors to account for biological variability. Cell culture chambers and tubing between valve and cell culture chamber and between cell culture chamber and sampling compartment were filled with M1 Macrophage Generation Medium DXF. The valve was rinsed and filled with fixation solution ((PBS Biochrom GmbH, Berlin, Germany) and the fixative compartment (syringe) was filled with 9.1 mM NaCl (Sigma-Aldrich Chemie GmbH, Steinheim, Germany), 0.0909mM EDTA (Sigma-Aldrich Chemie GmbH, Steinheim, Germany), 4.55 mM HEPES (Biochrom GmbH, Berlin, Germany) and 0.05% (*v*/*v*) formaldehyde in PBS (Biochrom GmbH, Berlin, Germany)). Assembly of the hardware (flight- and ground modules) was done on heating mats set to 36.5 °C to prevent the cells from cooling down. After completion of the assembly, both modules were tempered by identical build-in heating systems that was set to 36.5 °C and was active until the cells were fixed during the flight. The temperature profile was tracked in the flight module, showing that the temperature was between 36.47 and 36.49 °C at all times between experiment loading and fixation of the cells.

The TEXUS-54 rocket launched at 10.30 a.m. and cell-supernatant separation took place automatedly on board as foreseen at L + 50 s (BL-sample) and L+375 s (µg sample). Supernatant separation of the samples in the ground module was performed in the laboratory parallel to the flight samples. Shortly before the payload landed on the ground (approximately 7 min after the second fixation), the temperature control was turned off. From then on, the flight module cooled down to the ambient temperature. The landing site was covered with snow, the air temperature was measured as 10–14 °C. The module contained a temperature control that guaranteed that the temperature did not decrease below 4 °C. To create comparable conditions in the ground module, it was placed in a refrigerator (4–8 °C).

The rocket payload was picked up from the landing zone by helicopter in the afternoon and the FLUFIX flight module was retrieved in the laboratory at 5 p.m. Immediately, the device was opened and the cell culture supernatant with excess fixative was transferred from the sampling compartment to sample vials. So as not to lose any cell culture supernatant that might not have been transferred, fluids from the cell culture chambers and in tubing leading from the cell culture chamber to the sampling compartment was also collected and later combined in silico by combining the signals, weighted by the volume per chamber. One hour later, the samples of the ground module were taken analogously, and samples were stored at −80 °C.

#### 4.2.5. Rocket Flight Profile

The rocket flight profile is described by Thiel et al. [31].

### 4.3. Metabolomic Analysis

Metabolites from both campaigns were extracted using 900 μL cold (−20 °C) 80% methanol containing 20 μL ribitol (0.2 mg mL^−1^ in water) and 10 μL 13C-sorbitol (0.2 mg mL^−1^ in water), which were added as internal standards for the normalization of metabolite abundances (for calculation of relative metabolite abundance (RMA)). After incubation at 21 °C for 10 min, the extract was centrifuged for 15 min at 25,000× *g*. For further analysis, 50 μL of the supernatant was dried in vacuo. The pellet was resuspended in 10 μL of methoxyaminhydrochloride (20 mg mL^−1^ in pyridine) and derivatized for 90 min at 37 °C. After the addition of 20 μL of BSTFA (N,O-Bis[trimethylsilyl]trifluoroacetamide) containing 5 μL retention time standard mixture of linear alkanes (n-decane, n-dodecane, n-pentadecane, n-nonadecane, n-docosane, n-octacosane, n-dotriacontane), the mix was incubated at 37 °C for further 45 min. A volume of 1 μL of each sample was injected into a GC-TOF-MS system (Pegasus HT, Leco, St Joseph, MO, USA). Samples were derivatized and injected by an autosampler system (Combi PAL, CTC Analytics AG, Zwingen, Switzerland). Helium acted as carrier gas at a constant flow rate of 1 mL min^−1^. Gas chromatography was performed with an Agilent GC (7890A, Agilent, Santa Clara, CA, USA) using a 30 m VF-5ms column with 10 m EZ-Guard column. The injection temperature of the CIS injector (CIS4, Gerstel, Mühlheim, Germany) increased with a rate of 12 °C s^−1^ from initially 70 °C to finally 275 °C. The transfer line and ion source were set to 250 °C. The initial oven temperature (70 °C) was permanently increased to a final temperature of 320 °C by 9 °C per minute. To avoid solvent contaminations, the solvent delay was set to 340 s. Metabolites that passed the column were released into the TOF-MS. The temperature of the transfer line connecting the GC and the TOF-MS, as well as the ion source where the metabolites got ionized and fractionated by a pulse of 70 eV, were set to 250 °C. Charged mass fragments travelled through the vacuum flight tube until they reached the mass detector. Each fragment has a specific time of flight, depending on its mass charge ratio (*m*/*z*), until impact on the detector. Mass spectra were recorded at 20 scans per second with an *m*/*z* scanning range of 35–800. Chromatograms and mass spectra were evaluated using the ChromaTOF 4.5 and the TagFinder 4.1 software [98]. For Texus-54, three technical replicates were recorded for each sample, while in the CELLBOX-PRIME mission, two technical replicates for each ground module compartment and no technical replicates for the flight modules were recorded. A blank sample was recorded analogously for both campaigns, with three technical replicates for the Texus-54 mission and six technical replicates for the CELLBOX-PRIME mission.

Peak annotation was performed based on the open access Golm Metabolome Database (GMD) [99].

### 4.4. Statistical Analysis

A schematic of the analysis workflow is provided in Figure 3. Compounds which could not be matched with annotated metabolites in the GMD were removed. In the CELLBOX-PRIME data, missing values were present, but no missing values occurred in the TEXUS-54 data. For the technical replicates of the ground modules, if one value out of two was missing, 1/5 of the lowest measured signal in the whole dataset was used if the other technical replicate had a value below 50 (close to the limit of detection). Otherwise, the value of the technical replicate with a successful measurement was taken. The mean of the two technical replicates was then computed for each compartment of each module. For CELLBOX-PRIME, the mean RMA weighted by volume recovered from each compartment was subsequently calculated for each metabolite. For TEXUS-54, the mean of the three technical replicates per sample was taken. The difference arises due to the different hardware, and thus different sampling locations, in the two missions (Figure 1). Thereafter, an additional filtering step was carried out, in which metabolites with missing values in at least two out of five flight samples or one out of three ground samples were removed, but only if all other RMAs of the respective metabolite in the group with the missing values were above 50. Independently of this criterion, metabolites were removed if more than half the values were missing in both groups. After this filtering step, five metabolites still showed one missing value each. These were replaced with the mean. The latter step was also applied to the six blank samples (without prior filtering). If a metabolite in the blanks had only missing values, 1/5 of the minimal recorded value in the whole dataset was used. The subsequent data analysis steps, except where otherwise denoted, were conducted using R version 4.0.2. The session information is supplied in Supplementary Figure S4. To improve comparability between datasets, the CELLBOX-PRIME data was scaled by division with a scalar to possess the same mean as the TEXUS-54 data. Thereafter, the following problematic metabolites were removed: Sucrose, as it formed part of the fixation solution (only CELLBOX-PRIME); Nonadecane, as it was part of the reference mixture; Lactic acid and Phosphoric acid as they are involved in a multitude of cellular processes and changes are hard to interpret; Benzoic acid, Diisopropylamine, Edetic acid and Oxamide, as their natural occurrence in humans was not documented in the Human Metabolome Database (HMDB) [100] or the Small Molecule Pathway Database (SMPDB) [101]. Then, principal component analysis (PCA) was carried out on centered, unscaled data. Heat maps were generated on data standard-scaled on metabolites and subsequently scaled to [−1,1] by multiplication with the reciprocal of the maximum RMA. Hierarchical clustering was done using the complete linkage method. The unsupervised analysis allowed identification of sample FM.002 (CELLBOX-PRIME) as a potential outlier, which was omitted from downstream analysis. For CELLBOX-PRIME, a two-sample t-test with unequal variance was employed to test for statistical significance of differences between the mean RMA of each metabolite in the flight modules compared to the mean RMA of each metabolite in the ground modules. For TEXUS-54, a single-sample t-test with unequal variance was used to test for statistical significance of differences between the abundance of each metabolite in flight hypg and flight µg-conditions, and the mean RMA in the ground modules, respectively. Due to the exploratory nature of this study and because the use of a non-parametric test leads to undesirably low p-value resolution at low replicate numbers, the t-test was chosen despite the potentially unfulfilled normality assumption. Within-dataset cluster maps of metabolites were generated using Python version 3.7.3. The average clustering method from seaborn package version 0.10.1 using Euclidean distance was employed for hierarchical clustering.

## Figures and Tables

**Table 1:** Metabolites and metabolite clusters that are highlighted in the discussion. Significance levels are highlighted for TEXUS-54 hypg, for TEXUS-54 µg, and for CELLBOX-Prime datasets. The upper significance band is defined as FDR < 0.35 (“++”), the lower as FDR < 0.5 (“+”).

	Selected Metabolites Occurring in All Datasets	TEXUS-54 hypg	TEXUS-54 µg	CELLBOX-PRIME
Yellow Cluster	3-Hydroxybutyric acid	+	++	++
	L-Cysteine		+	+
	L-Phenylalanine			+
	L-Valine		+	+
	Threose		+	+
Pink Cluster	L-Arginine	++	++	
	myo-Inositole	++	++	
	L-Methionine			+
	L-Proline	++	++	+
	L-Aspartic Acid			
	Glycine		+	++
	Ketoleucine		++	++
	Ornithine	+	+	

## Data Availability

Data available in a publicly accessible repository. The data presented in this study are openly available in Dryad open repository name at [doi], reference number [reference number]. (confirmation pending).

## Supplementary Materials

Figure S1: Faceted bar plots for TEXUS-54 hypg vs. ground control, Figure S2: Full clustering correlation plot for both experiments, and Figure S3: Intra-experiment correlation plot for the two TEXUS-54 comparisons can be found at https://www.mdpi.com/article/10.3390/ijms22136752/s1.

## Abbreviations

Airbus DS	Airbus Defense and Space
TEXUS	German: Technologische Experimente unter Schwerelosigkeit
DLR	German Aerospace Center
MORABA DLR	Mobile Rocket Base
SSC	Swedish Space Cooperation
