# Supplementary material for: Metabolic Dynamics in Short- and Long-Term Microgravity in Human Primary Macrophages
Source: Int J Mol Sci. 2021 Jun 23;22(13):6752. doi: 10.3390/ijms22136752 (PMC8269311; doi:10.3390/ijms22136752)
Supplement: Supplementary file 1 [file ijms-22-06752-s001.zip › ijms-1259530-supplementary.pdf]

Supplementary Figure S1. Faceted bar plots for TEXUS-54 hypg vs ground control. Only metabolites with FDR ≤ 0.5 are shown. Each metabolite is shown with an individual relative metabolite abundance (RMA) scale. Ground samples are pooled from two biological samples, mean ± standard deviation is shown. +: FDR ≤ 0.5; ++: FDR ≤ 0.35.

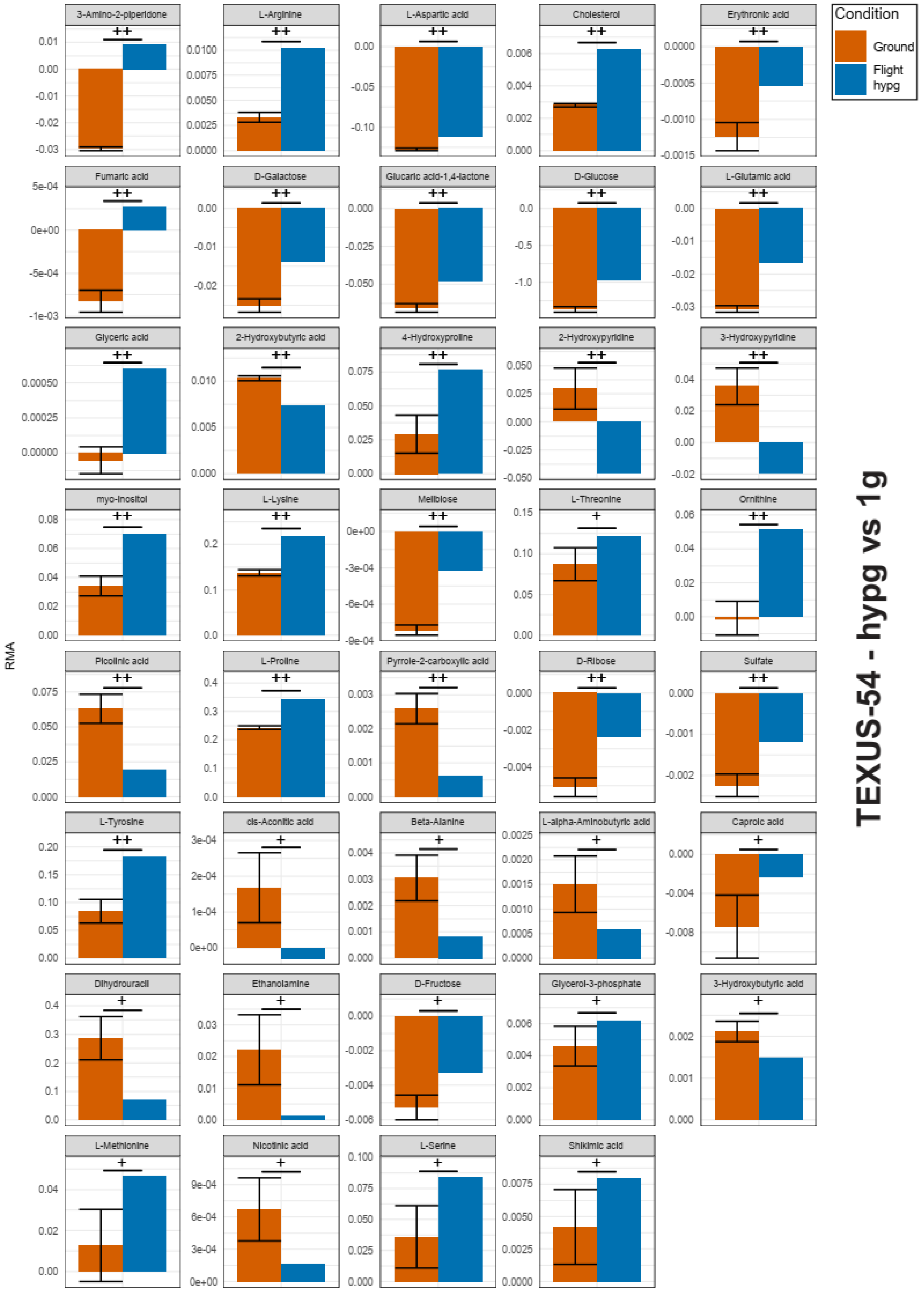

Supplementary Figure S2. Full clustering correlation plot for both experiments. Metabolites that are present in both datasets are indicated by bold metabolite names. Clustering trees are shown for each experiment. Sorting is based on clustering strength separately for each experiment; therefore metabolites are not in the same order. **A** Clustering heatmap for TEXUS-54 μg vs ground control. **B** Clustering heatmap for CELLBOX-PRIME flight vs ground.

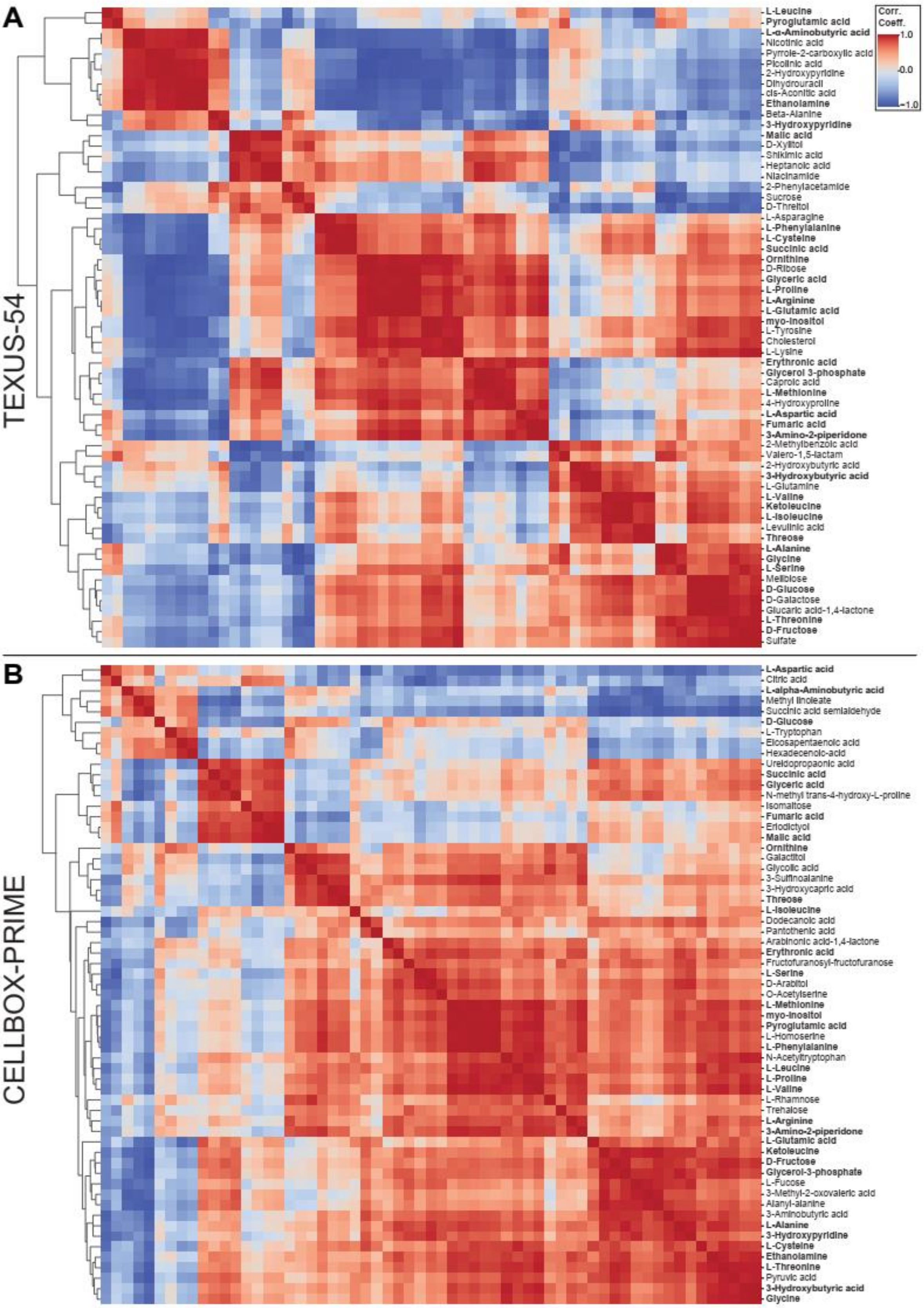

Supplementary Figure S3. Intra-experiment correlation plot for the two TEXUS-54 comparisons. Fold changes for each comparison are displayed as coordinates. The color indicates if the false discovery threshold is below the indicated value for both experiments. For most metabolites, the metabolic reactions towards altered gravity are similar, only a few metabolites lie in the upper left or the lower right quadrant.

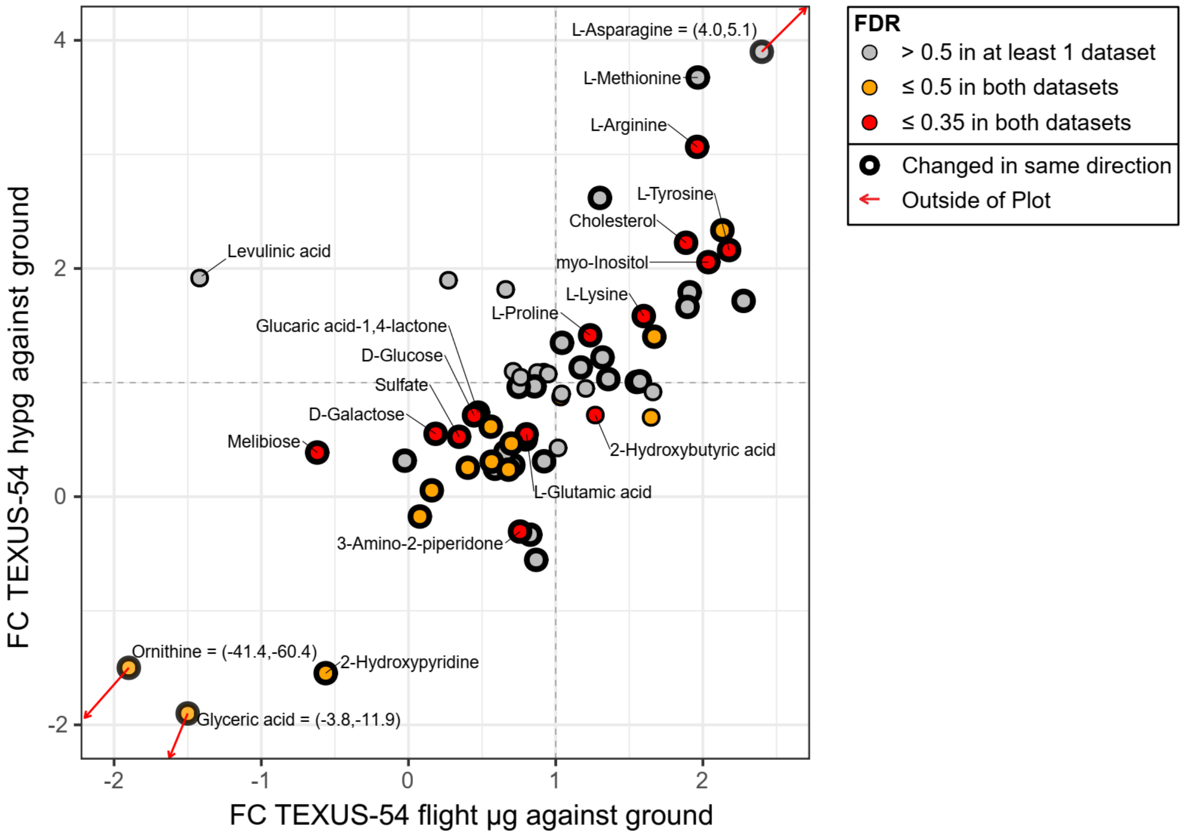

R session info figures publication Tx54 and CELLBOX-PRIME

Authors: Timothy Bradley, Christian Vahlensieck

Version: September 20, 2020

Description: R session info of the session used for generation of the publication figures.

```
R version 4.0.2 (2020-06-22)
Platform: x86_64-pc-linux-gnu (64-bit)
Running under: Debian GNU/Linux 10 (buster)

Matrix products: default
BLAS:   /usr/lib/x86_64-linux-gnu/openblas/libblas.so.3
LAPACK: /usr/lib/x86_64-linux-gnu/libopenblasp-r0.3.5.so

locale:
 [1] LC_CTYPE=en_US.UTF-8       LC_NUMERIC=C
 [3] LC_TIME=en_US.UTF-8        LC_COLLATE=en_US.UTF-8
 [5] LC_MONETARY=en_US.UTF-8    LC_MESSAGES=en_US.UTF-8
 [7] LC_PAPER=en_US.UTF-8       LC_NAME=C
 [9] LC_ADDRESS=C               LC_TELEPHONE=C
[11] LC_MEASUREMENT=en_US.UTF-8 LC_IDENTIFICATION=C

attached base packages:
[1] parallel  grid      stats     graphics  grDevices utils     datasets
[8] methods   base

other attached packages:
 [1] extrafont_0.17       ggsignif_0.6.0       ComplexHeatmap_2.4.3
 [4] Cairo_1.5-12.2       plyr_1.8.6           gplots_3.0.4
 [7] plotly_4.9.2.1       RColorBrewer_1.1-2   cowplot_1.0.0
[10] pcaMethods_1.80.0    Biobase_2.48.0       BiocGenerics_0.34.0
[13] plotrix_3.7-8        scales_1.1.1         EnhancedVolcano_1.6.0
[16] ggrepel_0.8.2        gridExtra_2.3        ggforce_0.3.2
[19] ggpubr_0.4.0         forcats_0.5.0        stringr_1.4.0
[22] dplyr_1.0.2          purrr_0.3.4          readr_1.3.1
[25] tidyr_1.1.2          tibble_3.0.3         ggplot2_3.3.2
[28] tidyverse_1.3.0

loaded via a namespace (and not attached):
 [1] colorspace_1.4-1    rjson_0.2.20        ellipsis_0.3.1
 [4] rio_0.5.16          circlize_0.4.10     IRdisplay_0.7.0
 [7] GlobalOptions_0.1.2 base64enc_0.1-3     fs_1.5.0
[10] clue_0.3-57         rstudioapi_0.11     farver_2.0.3
[13] fansi_0.4.1         lubridate_1.7.9     xml2_1.3.2
[16] polyclip_1.10-0     IRkernel_1.1.1      jsonlite_1.7.1
[19] broom_0.7.0         Rttf2pt1_1.3.8      cluster_2.1.0
[22] dbplyr_1.4.4        png_0.1-7           compiler_4.0.2
[25] httr_1.4.2          backports_1.1.9     assertthat_0.2.1
[28] lazyeval_0.2.2      cli_2.0.2           tweenr_1.0.1
[31] htmltools_0.5.0     tools_4.0.2         gtable_0.3.0
[34] glue_1.4.2          Rcpp_1.0.5          carData_3.0-4
[37] cellranger_1.1.0    vctrs_0.3.4         gdata_2.18.0
[40] extrafontdb_1.0     openxlsx_4.1.5      rvest_0.3.6
[43] lifecycle_0.2.0     gtools_3.8.2        rstatix_0.6.0
[46] MASS_7.3-52         hms_0.5.3           curl_4.3
[49] stringi_1.4.6       caTools_1.18.0      zip_2.1.1
[52] shape_1.4.4         repr_1.1.0          rlang_0.4.7
[55] pkgconfig_2.0.3     bitops_1.0-6        evaluate_0.14
[58] htmlwidgets_1.5.1   labeling_0.3        tidyselect_1.1.0
[61] magrittr_1.5        R6_2.4.1            generics_0.0.2
[64] pbdZMQ_0.3-3        DBI_1.1.0           pillar_1.4.6
[67] haven_2.3.1         foreign_0.8-80      withr_2.2.0
[70] abind_1.4-5         modelr_0.1.8        crayon_1.3.4
[73] car_3.0-9           uuid_0.1-4          KernSmooth_2.23-17
[76] GetoptLong_1.0.2    readxl_1.3.1        data.table_1.13.0
[79] blob_1.2.1          reprex_0.3.0        digest_0.6.25
[82] munsell_0.5.0       viridisLite_0.3.0
```
